# HOXA5 inhibits the proliferation of extrahepatic cholangiocarcinoma cells by enhancing MXD1 expression and activating the p53 pathway

**DOI:** 10.1038/s41419-022-05279-6

**Published:** 2022-09-27

**Authors:** Fei Xiong, Wenzheng Liu, Xin Wang, Guanhua Wu, Qi Wang, Tong Guo, Wenhua Huang, Bing Wang, Yongjun Chen

**Affiliations:** 1 Department of Biliary‑Pancreatic Surgery, Tongji Hospital, Tongji Medical College, Huazhong University of Science and Technology, Wuhan, Hubei China; 2 Departement of Pediatric Surgery, Wuhan Children’s Hospital, Tongji Medical College, Huazhong University of Science and Technology, Hubei Wuhan, China; 3 Department of Emergency, Tongji Hospital, Tongji Medical College, Huazhong University of Science and Technology, Wuhan, Hubei China

**Keywords:** Cancer, Diseases

## Abstract

Homeobox A5 (HOXA5) is a transcription factor in mammalian and can regulate cell differentiation, proliferation, and apoptosis as well as tumorigenesis. However, little is known on whether and how HOXA5 can regulate the malignant behaviors of cholangiocarcinoma. The methylation levels of HOXA5 were evaluated by methylation microarray and bisulfite sequencing PCR. HOXA5 expression in tissue samples was examined by immunohistochemistry and Western blot. The proliferation of tumor cells was assessed by CCK-8, EdU, and nude mouse tumorigenicity assays. The invasion, apoptosis and cell cycling of tumor cells were evaluated by Wound healing assay and flow cytometry. The interaction between HOXA5 and the MXD1 promoter was examined by CUT & Tag assay, luciferase reporter assay and chromatin immunoprecipitation. Hypermethylation in the HOXA5 promoter down-regulated HOXA5 expression in extrahepatic cholangiocarcinoma (ECCA) tissues, which was correlated with worse overall survival. HOXA5 overexpression significantly inhibited the proliferation and tumor growth. HOXA5 overexpression enhanced MXD1 expression by directly binding to the MXD1 promoter in ECCA cells. MXD1 overexpression inhibited the proliferation and tumor growth while MXD1 silencing abrogated the HOXA5-mediated proliferation inhibition. HOXA5 overexpression increased p53 protein expression in an MXD1-dependent manner. HOXA5 and MXD1 acted as tumor suppressors to inhibit the mitosis of ECCA cells by enhancing the p53 signaling. Our findings may uncover molecular mechanisms by which the HOXA5/MXD1 axis regulates the progression of ECCA, suggesting that the HOXA5/MXD1 may be therapeutic targets for ECCA.

## Introduction

Cholangiocarcinoma is a malignant tumor that originates from biliary epithelial cells and is characterized by a high degree of malignancy and concealment of disease with atypical early symptoms. In recent years, the incidence rate of cholangiocarcinoma is increasing yearly [1]. Because of its low rate of resection and insensitive to traditional radiotherapy and chemotherapy, the prognosis of cholangiocarcinoma is very poor [2]. Therefore, it is of great significance to understand the pathogenesis of cholangiocarcinoma and which factors regulate the growth of cholangiocarcinoma for the development of personalized targeted therapies.

Homeobox A5 (HOXA5) is a transcription factor in mammalian and can regulate cell differentiation, proliferation, and apoptosis as well as tumorigenesis [3]. HOXA5 can attenuate the growth and progression of cervical cancer and colorectal cancer by inhibiting the Wnt/β-catenin signaling [4, 5]. Furthermore, HOXA5 acts as a tumor suppressor to inhibit the angiogenesis of hepatocellular carcinoma and induces the apoptosis of lung cancer cells [6, 7]. However, little is known on whether and how HOXA5 can regulate the malignant behaviors of cholangiocarcinoma.

MAX dimerization protein 1 (MXD1, also known as Mad1) is a basic helix-loop helix-leucine-zipper (bHLH/LZip) protein in the MYC/MXD/MAX family and can functionally compete with MYC for MAX binding to form a transcription repressor to down-regulate cell transformation, differentiation, proliferation and apoptosis [8]. Down-regulated MXD1 expression supports the survival, invasion, and metastasis of pancreatic, breast and gastric cancer cells [9–11]. Up-regulated MXD1 expression inhibits the proliferation of tumor cells stimulated by many factors, such as exogenous Vitamin D3 and Mps1 inhibitors [12, 13]. However, there is no information on whether HOXA5 can regulate MXD1 expression and its downstream signaling in cholangiocarcinoma.

The current study explored the methylation status of the HOXA5 promoter in extrahepatic cholangiocarcinoma (ECCA) and examined how HOXA5 overexpression modulated the malignant behaviors of ECCA cells. Subsequently, we explored the potential targets of HOXA5 and how their changes in expression levels affected the malignant behaviors of ECCA cells. We found that hypermethylation in the HOXA5 promoter led to down-regulated HOXA5 expression in ECCA. HOXA5 overexpression inhibited the proliferation of ECCA cells by up-regulating MXD1 and downstream p53 expression. Furthermore, HOXA5 was directly bound to the MXD1 promoter and enhanced the downstream luciferase expression in luciferase reporter assays. Our novel data indicated that HOXA5 acted as a tumor suppressor to inhibit the proliferation of ECCA cells by enhancing the MXD1/p53 signaling. Therefore, our findings may uncover new molecular mechanisms underlying the pathogenesis of ECCA and shed light on therapeutic targets for development of new therapies for ECCA.

## Materials and methods

### Special antibodies

Antibodies included anti-HOXA5 (HPA029319), anti-MXD1 (HPA001599, Atlas Antibodies, Stockholm, Sweden); anti-Ki67 (9027 S), anti-MYC (9402 S) and anti-Flag (14793 S, Cell Signaling Technology, Leiden, the Netherlands); anti-α-Tubulin (11224-1-AP), anti-MXD1 (17888-1-AP), anti-NASP (11323-1-AP), anti-BCL-2 (12789-1-AP), anti-E-Cadherin (20874-1-AP), anti-N-Cadherin (22018-1-AP), anti-p53 (10442-1-AP) and anti-Vimentin (60330-1-Ig, Proteintech, Chicago, USA); anti-CDK2 (BM3926), anti-CDK4 (BM1572), anti-MCM6 (M02755), anti-PCNA (BM0104), anti-AIFM1 (M01571-1, BOSTER, Wuhan, China); and normal rabbit IgG (A7016, Beyotime, Shanghai, China).

### Bioinformatics

The levels of gene mRNA transcripts from the microarrays of cancerous and para-cancerous tissues (GSE3189, GSE13898, GSE32863, GSE9348, GSE13507, GSE3744, GSE25099, GSE13519, GSE9750 and GSE6338) and methylation microarrays (GSE49656 and GSE38860) (Table S1) were obtained from Gene Expression Omnibus (GEO) database (https://www.ncbi.nlm.nih.gov/geo/) and analyzed in the Oncomine database (https://www.oncomine.org/). The data were normalized and statistically analyzed. The expression of Homeobox (HOX) genes was retrieved from GEPIA database (http://gepia.cancer-pku.cn/) [14].

The nucleotide sequences of the MXD1 and HOXA5 promoters were obtained from Gene database (https://www.ncbi.nlm.nih.gov/gene/) for analyzing the methylation level and predicting the potential binding sites. The matrix profile MA0158.2 of HOXA5 was downloaded and the potential binding sites in the MXD1 promoter were predicted using JASPAR (https://jaspar.genereg.net/) [15]. The fold-change value was obtained from the logarithm (logFC) analysis. The differentially expressed genes (DEGs) were defined when adjusted *P* < 0.05 and |logFC | ≥1. The potentially biological functions and pathways of DEGs were analyzed using the Database for Annotation, Visualization and Integrated Discovery (DAVID) (https://david.abcc.ncifcrf.gov/), including Gene Ontology (GO), Kyoto Encyclopedia of Genes and Genomes (KEGG) and UCSC Transcription Factor Binding Site (UCSC TFBS) [16, 17]. The results were displayed by R package “GOplot” in R 3.6.3 [18].

### Tissue samples

Twelve surgical ECCA, twelve surgical intrahepatic cholangiocarcinoma (ICCA) and their para-carcinoma non-tumor tissues were collected in the Department of Biliary-Pancreatic Surgery, Tongji Hospital of Huazhong University of Science and Technology, Wuhan, China. The experimental protocols were approved by the Ethical Committee of Tongji Hospital of Huazhong University of Science and Technology. Individual patients signed informed consent.

### Tissue microarray and immunohistochemistry (IHC)

The levels of HOXA5 protein expression in different cholangiocarcinoma and control tissues were examined by IHC. Briefly, the paraffin-embedded tissue microarray samples were obtained from Outdo Biotech, Shanghai, China, and included 17 ECCA, 11 extrahepatic bile duct, 91 ICCA and 31 intrahepatic bile duct samples. The tissue samples were deparaffinized, rehydrated and subjected to antigen retrieval. After being blocked with 5% bovine serum albumin, the tissue sections were probed with primary antibodies overnight at 4 °C and incubated with horseradish peroxidase (HRP)-labeled secondary antibodies, followed by visualizing with DAB staining. The intensity of antibody staining (0: negative, 1: weak, 2: moderate and 3: strong) and percentage of positive cells (0: negative, 1: 1–25%, 2: 26–50%, 3: 51–75% and 4: 76–100%) were scored in a blinded manner. The product of intensity score and percentage of positive cell score served as an IHC score. Similarly, xenograft tumor tissue sections (4 µm) were analyzed by IHC.

### Cell culture and treatment

Human ECCA TFK-1 and EGI-1 cells and human embryonic kidney HEK-293T cells were authenticated, maintained, and kept free of contamination in our laboratory. TFK-1, EGI-1 cells and HEK-293T cells were cultured in RPMI 1640 and DMEM supplemented with 10% fetal bovine serum, 100 U/mL penicillin and 100 μg/mL streptomycin (complete medium) at 37 °C in a humidified incubator of 5% CO_2_, respectively. Some cells were treated with 25 μM decitabine (DCA) (#S1200, Selleck Chemicals, Shanghai, China) for 3 days with daily change in complete medium.

### Lentivirus transduction

Lentiviruses for Flag-HOXA5 overexpression (LV-HOXA5, NM_019102), Flag-HOXA5 mutant overexpression (LV-HOXA5 Mut) and Flag-MXD1 overexpression (LV-MXD1, NM_001202513), MXD1 silencing (LV-shMXD1-1, GCACCAGCATCAAGAGAATAA and LV-shMXD1-2, GCCAAATTGCACATAAAGAAA) and their corresponding negative controls were constructed and produced by Genechem, Shanghai, China. TFK-1 and EGI-1 cells were transduced with specific lentiviruses at a multiplicity of infection (MOI) of 20 in the presence of HistransG (Genechem) for 24 h. The cells were cultured in complete medium for two days and treated with 1 μg/ml of puromycin for the establishment of stable cell lines.

### RNA extraction, quantitative real-time PCR (RT-qPCR) and microarray

Total RNA was extracted from individual cell samples using RNA Extraction Reagent (Vazyme, Nanjing, China) and reversely transcribed into cDNA using the HiScript III RT SuperMix for qPCR (+gDNA wiper) (Vazyme), according to the manufacturer’s instructions. The RT-qPCR reactions were performed in triplicate with the ChamQ Universal SYBR qPCR Master Mix (Vazyme) and specific primers (Table S2) in the iQ5™ quantitative PCR detection system (Bio-Rad, Richmond, CA, USA). The data were analyzed using 2^−ΔΔCt^ method.

Total RNA was also used for analysis of Human OneArray Gene Expression Profiling (Phalanx Biotech, Hsinchu City, China Taiwan) using log2 |FC | ≥ 1 and adjusted *P* < 0.05 to identify DEGs. After hybridization, the arrays were washed, scanned and the gene expression results were extracted by DNA Microarray Scanner G2565B (Agilent, California, USA), according to the manufacturer’s instructions. Raw fluorescence intensity values were normalized and log-transformed using GeneSpring GX 10 software (Agilent).

### Western blot

The relative levels of protein expression in tissue and cell samples were quantified by Western blot. Briefly, freshly tissue samples were homogenized and cell samples were lyzed in RIPA buffer containing the Protease Inhibitor Cocktail (Roche, Basel, Switzerland). After being centrifuged, the concentrations of total proteins were determined. Individual lysate samples (30 µg/lane) were separated by sodium dodecyl sulfate polyacrylamide gel electrophoresis (SDS-PAGE) and transferred onto nitrocellulose membranes (Millipore, Bedford, USA). After being blocked in 5% skimmed dry milk in TBST, the membranes were incubated with diluted primary antibodies overnight at 4 °C. The bound antibodies were reacted with HRP-conjugated anti-rabbit and anti-mouse IgG (BOSTER) and visualized by the ECL (Thermo Fisher Scientific, Waltham, USA). The data were analyzed by Image Lab software (Bio-Rad). The result was uploaded as supplemental material.

### CUT & Tag, library construction and DNA sequencing

The binding pattern of HOXA5 in the genome of TFK-1 cells was examined by CUT & Tag assay using the Hyperactive Universal CUT&Tag Assay Kit (Vazyme), according to the manufacture’s instruction. Briefly, 1 × 10^6^ TFK-1 cells were harvested and their nuclei were extracted. The extracted nuclei were immobilized with activated concanavalin A‐coated magnetic beads and reacted with anti-Flag antibodies (1:50) in 100 μL DIG buffer overnight at 4 °C. After being washed, the bound antibodies were reacted with secondary antibodies (1:50) for 1 h at room temperature and incubated with 100 μl Hyperactive pA/G-Transposon adapter complex (0.04 μM) for 1 h at room temperature. The precipitated immunocomplex was diluted in Trueprep Tagment Buffer L and after proteinase K digestion, the DNA fragments were extracted and stored at −80 °C. Subsequently, a DNA library was constructed using the TruePrep Index Kit V2 (Vazyme). The DNA fragments were amplified by PCR at 72 °C for 3 min, 95 °C for 3 min, and subsequent 10 cycles of 98 °C for 10 s and 60 °C for 5 s, 72 °C for 1 min and hold at 4 °C. The library was extracted by VAHTS DNA Clean Beads (Vazyme). The specific gene sequences were analyzed with DNA sequencing in an Illumina PE150 platform (Illumina) by HaploX, Shangrao, China.

### Luciferase reporter assay

The luciferase reporter vector carrying whole length of the promoter of MXD1 (Promoter-MXD1), plasmid for the expression of HOXA5 (TFs-HOXA5) and the negative control vector (Promoter-NC and TFs-NC) were constructed by Genechem. The vectors carrying four mutated points respectively were constructed by Genechem. HEK293T cells (10^5^ cells/well) were cultured in 24-well plates and transfected with luciferase reporter vector, TFs-HOXA5 or controls using X-tremegene HP (Roche) for 48 h. The luciferase activities in different groups of cells were determined in the Dual-Luciferase Assay system (Promega, Wisconsin, USA), according to the manufacturer’s instructions.

### Chromatin immunoprecipitation (ChIP)

The different groups of cells (10^7^ cells/sample) were fixed with 1% formaldehyde and treated with 10% glycine. Subsequently, the sonicated chromatin was immunoprecipitated with primary antibodies and the formed immunocomplex was precipitated with protein A + G Agarose beads (Med Chem Express, Shanghai, China). The contents of DNA in the immunocomplex samples were amplified by PCR using the primers (Table S2).

### Proliferation assay

The proliferation of different groups of cells was quantified by CCK-8 assay. Briefly, each group of cells (1.5 × 10^3^ cells/well) was grown in 96-well plates for 0, 24, 48, 72, or 96 h. Each well was added with 10 μL of CCK8 in Cell Counting Kit (Yeasen, Shanghai, China) and incubated for another two hours. The absorbance at 450 nm in individual wells of cells was measured using a MULTISKAN FC microplate reader (Bio-Rad).

Similarly, the proliferation of each group of cells was also quantified by EdU assay using Cell-Light EdU Apollo567 In Vitro Kit (100 T) and Cell-Light EdU Apollo488 In Vitro Kit (100 T) (Ribobio, Guangzhou, China), according to the manufacturer’s instruction. Briefly, each group of cells (5 × 10^4^ cells/well) was cultured in 24-well plates for 3 days. The EdU incorporation in individual cell samples was observed and photographed under a confocal laser scanning microscope (Leica Biosystems, CA, USA).

### Wound healing assay

The different groups of ECCA cells were cultured in 6-well plates until reaching almost 100% of confluency. The monolayer of cells was wounded using a pipette tip of 20 μL and cultured in serum-free RPMI 1640 for 24 h. The wound healing was observed and photographed under an inverted microscope. The wound healing of ECCA cells was evaluated as Cell wound healing rate = (Scratched area (0 h)–the remaining area at 24 h)/Scratched area (0 h)).

### Flow cytometry

The percentages of apoptotic cells and the cells in different phases of cell cycling in individual groups were examined by flow cytometry using the Annexin V-APC/7-AAD apoptosis and cell cycle staining kits (Multi Science, Hangzhou, China), respectively. Briefly, individual groups of cells (1 × 10^6^ per sample) were stained with Annexin V-APC/7-AAD or DNA staining solution. After being washed, the percentages of cells in different groups were quantified by flow cytometry in the Becton-Dickinson FACScan System (Franklin Lakes, NJ, USA).

### Methylation microarray

TFK-1 and non-tumor bile duct epithelial samples were lysed and homogenized, respectively. Their genomic DNA was extracted using the DNeasy Blood & Tissue Kit (Qiagen, Hilden, Germany), according to the manufacturer’s instruction. Individual DNA samples (11 µg each) were sonicated for 10 cycles of sonication for 30 s and rested for another 30 s. The methylated DNA was enriched by immunoprecipitation, as described previously [19]. The precipitated and reference DNA were extracted with phenol-chloroform. The DNA samples (1–2 μg each) were combined and hybridized to NimbleGen HG18CpG promoter (Roche, Basel, Switzerland), according to the manufacturer’s instruction. The microarrays were scanned with NimbleScanTM2.2 microarray scanner and differential methylation peaks between two groups of samples were analyzed using the SignalMap software v1.9.

### Bisulfite sequencing PCR (BSP)

The bisulfate modified DNA was amplified with forward and reverse primers for target genes. The PCR products (1 µl each) were cloned into pCR-TOPO using the TOPO TA cloning kit (Invitrogen, California, USA), according to the manufacturer’s instructions. After being transformed into One Shot TOP 10 chemically competent cells, the plasmids in 10 grown colonies were extracted and sequenced using T7 or M13 forward and reverse primers.

### Xenograft models

Forty-five female Balb/c nude mice (4–6 weeks old) were obtained from Charlies River, Beijing, China. The mice were randomized into each group (*n* = 5 per group), based on our preliminary sample size analysis. Individual mice that failed to develop tumors subcutaneously were excluded from analysis. No blinding was done. Individual mice were injected subcutaneously with 2 × 10^6^ each group of TFK-1 cells into their axillary cavity. Two weeks after the injection, the mice were euthanized. Their subcutaneous tumors were excised, measured and used for IHC. The tumor volumes were calculated with the following equation: tumor volume (mm^3^) = 0.5 × length × width^2^. All the experiments were performed, according to the protocol approved by the Ethical Committee of Tongji Hospital of Huazhong University of Science and Technology.

### Statistical analysis

All the data are representative images of each group or presented by means ± standard error of mean (SEM) from three separate experiments. The distribution, variation and variance of data from different groups were estimated by GraphPad Prism 8.0 (GraphPad). The difference between groups was compared by Student’s *t* test and the difference among groups was analyzed by ANOVA and post hoc Bonferroni multiple comparisons test. The survival of each group of subjects was estimated by Kaplan–Meier method and analyzed by log-rank test. The relationship between variants was analyzed by Pearson correlation coefficient (Pearson r). A two-tailed *P*-value of <0.05 was considered statistically significant.

## Results

### Hypermethylation in the HOXA5 promoter exists in ECCA

**Fig. 1:**
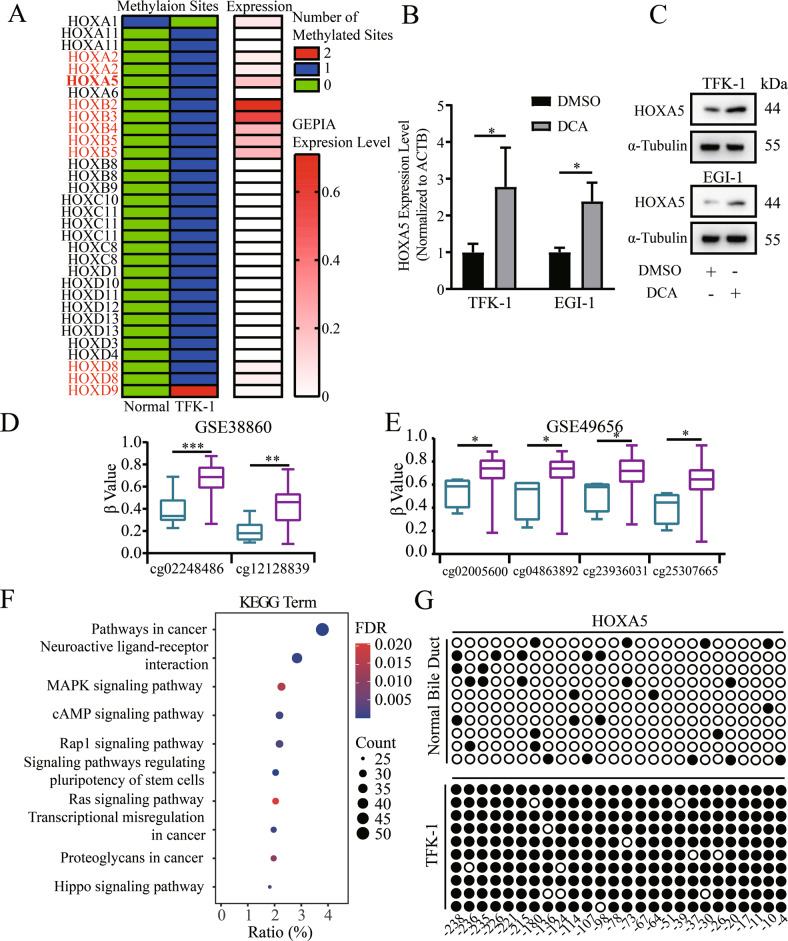
The methylation profile of the HOXA5 gene in ECCA. **A** Heatmap analysis of the methylation and expression level of the HOX genes. The gradual changes from red to white represent changes in gene expression from high to low. Green, no CpG island. Blue, 1 CpG island. Red, 2 CpG islands. **B**, **C** RT-qPCR and Western blot analyses of HOXA5 expression in ECCA cells following treatment with, or without, DCA. **D**, **E** Analysis of the methylation profile of the HOXA5 gene in GSE49656 and GSE38860 of GEO database. The purple box represents the cancer samples and the blue box represents the non-tumor samples. **F** Bubbles plotted the KEGG analysis of the methylation levels of differential genes. The gradual changes from red to blue represent the changes in high to low FDR. The sizes of bubbles represent the numbers of genes enriched in certain terms. **G** The methylation profile of the HOXA5 gene from the BSP results. The black solid represents methylated sites and the hollow points represent unmethylated sites. *P < 0.05. **P < 0.01. ***P < 0.001.

Abnormal DNA methylation in the promoter of tumor suppressor genes contributes to the development of malignant tumors [20]. Accordingly, we explored the methylation profile of human ECCA by a methylation microarray in TFK-1 and non-tumor bile duct epithelial samples. We found that 1082 CpG islands of known genes were hypomethylated and 1697 were hypermethylated in cholangiocarcinoma cells, related to non-tumor bile duct epithelial samples. Interestingly, most genes in the HOX gene family, including HOXA5, but not HOXA1, were hypermethylated (Fig. 1A). To further narrow down the potential key HOX genes, we retrieved the data about the expression of these HOX genes in GEPIA database and found that only seven HOX genes could be detected in bile duct tissues (Fig. 1A, Fig. S1A). To explore the relation between methylation and gene expression, we treated TFK-1 and EGI-1 cells with decitabine (DCA, 25 μM) for 72 h. We found that decitabine treatment increased the relative levels of HOXA5 mRNA transcripts and protein expression in both cell lines (Fig. 1B, C), but up-regulated HOXA2 and HOXB4 only in TFK-1 cells, not in EGI cells (Fig. S1B). Accordingly, we chose HOXA5 for subsequent experiments. Given that HOXA9 and HOXB13 expression can be regulated by DNA methylation [21, 22], and HOXA5 encodes a transcription factor to regulate the proliferation, differentiation and survival of cells [4, 5], we analyzed the levels of HOXA5 methylation in GSE49656 and GSE38860. The levels of HOXA5 promoter methylation in cholangiocarcinoma tissues were significantly higher than those in non-tumor tissues (Fig. 1D, E). KEGG analysis revealed that the hypermethylated genes were enriched in some terms involved in regulating cell proliferation (e.g., “MAPK signaling pathway”, “Ras signaling pathway” and “Hippo signaling pathway”) (Fig. 1F, Table S3). Actually, BSP indicated that the levels of HOXA5 promoter methylation in TFK-1 cells increased, compared with that in the control cells (Fig. 1G). Hence, hypermethylation in the HOXA5 promoter down-regulated its expression in ECCA, probably contributing to the pathogenesis of ECCA.

### Down-regulated HOXA5 expression is associated with worse overall survival (OS) of patients with ECCA

**Fig. 2:**
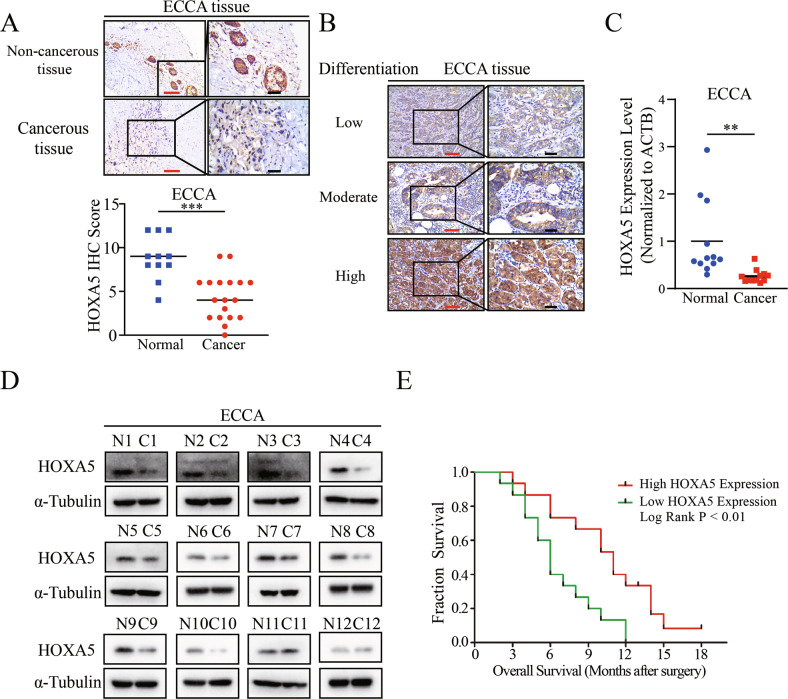
HOXA5 expression in ECCA tissues. **A** Immunohistochemistry analysis of HOXA5 expression in ECCA and non-tumor tissues. **B** Immunohistochemistry analysis of HOXA5 expression in different differentiation levels of ECCA from patients. **C**, **D** RT-qPCR and Western blot analyses of HOXA5 expression in ECCA and paired non-tumor tissues. *N* Non-tumor samples, *C* ECCA samples. **E** Lower levels of HOXA5 expression were associated with a worse OS of ECCA in this population. ***P < 0.001. **P < 0.01. Red bar, 100 μm. Black bar, 40 μm.

HOXA5, a tumor suppressor, is down-regulated in breast cancer, cervical carcinoma, and hepatocellular carcinoma [4, 7, 23]. To understand the expression pattern of HOXA5 in cholangiocarcinoma, we characterized the expression of HOXA5 in cholangiocarcinoma and controlled tissue arrays by IHC. The results revealed that HOXA5 was mainly expressed in the nucleus of cells (Fig. 2A). The levels of HOXA5 expression in non-cancerous tissues were significantly higher than that in ECCA tissues (Fig. 2A). The levels of HOXA5 expression were associated with the differentiation levels of ECCA (Fig. 2B). However, there was no significant difference in the levels of HOXA5 expression between the ICCA and non-cancerous tissues (Fig. S2A). Western blot and RT-qPCR analyses further validated that the levels of HOXA5 expression were down-regulated in ECCA tissues, related to the matched adjacent tissues, which was not observed in ICCA (Fig. 2C, D, Fig. S2B), suggesting that HOXA5 might be crucial for the pathogenesis of ECCA. Indeed, we found that lower levels of HOXA5 expression were associated with poor pathological differentiation and higher TNM stages of ECCA in this population (Table S4). More importantly, stratification indicated that lower levels of HOXA5 expression were associated significantly with a worse OS of ECCA patients (Fig. 2E). However, the levels of HOXA5 expression were not significantly correlated with the survival of ICCA patients (Fig. S2C).

To look paralleling evidence for the role of HOXA5 in the pathogenesis of ECCA, we retrieved the levels of HOXA5 mRNA transcripts in Oncomine database. Among 47 studies, 11 studies reported up-regulated HOXA5 mRNA transcripts and 36 with down-regulated HOXA5 mRNA transcripts in cancer tissues, related to the controls (Fig. S3A). The levels of HOXA5 transcripts ranked the lowest in breast cancer (15 studies), lung cancer (10 studies), colorectal cancer (6 studies), and melanoma (2 studies) and representative figures are shown in Fig. S3B–G, suggesting that HOXA5 may act as a tumor suppressor of cancer development, which is conserved in ECCA rather than ICCA.

### HOXA5 attenuates the malignant behaviors of ECCA cells by inducing cell cycle arrest and apoptosis

**Fig. 3:**
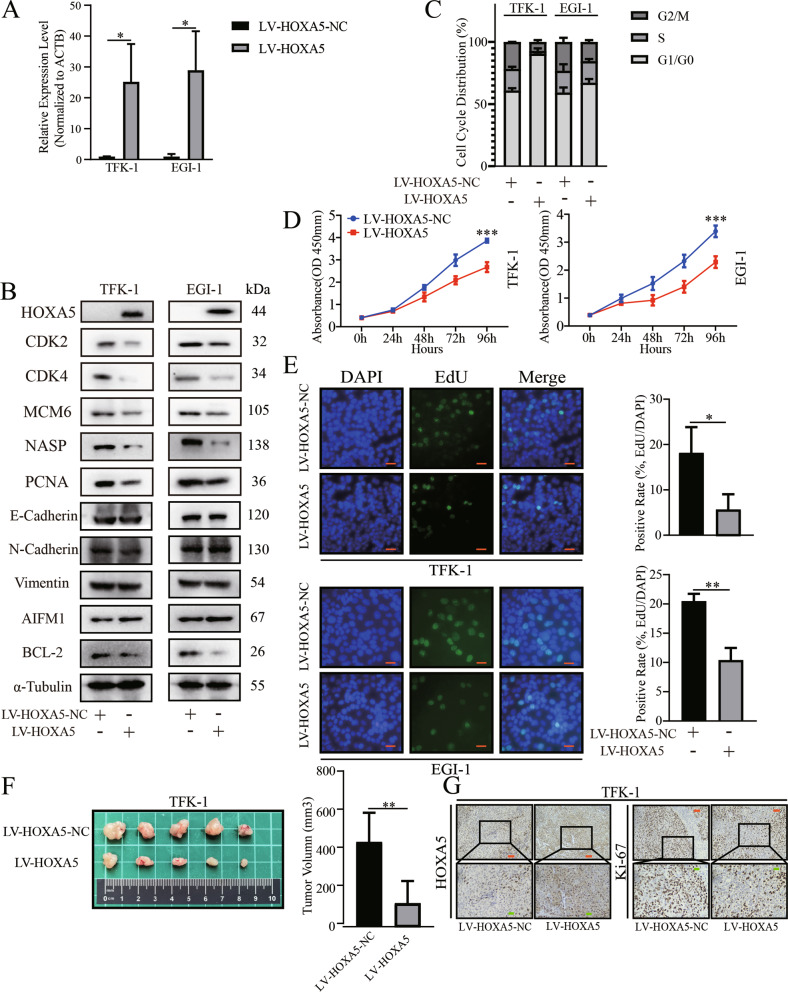
HOXA5 overexpression attenuates the malignant behaviors of ECCA cells. **A**, **B** RT-qPCR and Western blot analyses validated HOXA5 overexpression, which reduced the mitosis-related gene expression in ECCA cells. HOXA5 overexpression did not alter the EMT-related gene expression, but increased AIFM1, and decreased BCL-2 expression in ECCA cells. **C** HOXA5 overexpression induced cell cycle arrest in G1/G0 phase. **D**, **E** HOXA5 overexpression inhibited the proliferation of ECCA cells. **F** HOXA5 overexpression decreased the tumor volumes in mice. **G** Immunohistochemistry analysis of HOXA5 and Ki67 expression in xenograft tumors from nude mice. Data are representative images from three separate experiments. *P < 0.05. **P < 0.01. ***P < 0.001. Orange bar, 50 μm. Green bar, 20 μm.

Next, we evaluated the function of HOXA5 in TFK-1 and EGI-1 cells. First, very low levels of HOXA5 expression were detected in TFK-1 and EGI-1 cells (Fig. 3A, B). Comparison with the control cells, HOXA5 overexpression significantly reduced CDK2, CDK4, MCM6, NASP, and PCNA expression levels in TFK-1 and EGI-1 cells (Fig. 3B) and decreased the proliferation ability of TFK-1 and EGI-1 cells (Fig. 3C–E). Consistently, HOXA5 overexpression induced cell cycling arrest in G0/G1 phase in both cell lines (Fig. 3C). While HOXA5 overexpression did not significantly alter the wound healing of ECCA cells (Fig. S4A), it did significantly increase the frequency of apoptotic TFK-1 and EGI-1 cells (Fig. S4B). Consistently, HOXA5 overexpression increased AIFM1, but decreased BCL-2 expression levels in TFK-1 and EGI-1 cells (Fig. 3B). However, HOXA5 overexpression did not significantly change E-cadherin, N-cadherin and Vimentin expression levels in TFK-1 and EGI-1 cells (Fig. 3B). HOXA5 overexpression significantly decreased the volumes of implanted TFK-1 tumors and their Ki67 expression in vivo (Fig. 3F, G). Collectively, HOXA5 overexpression attenuated the malignant behaviors of ECCA cells in vitro and in vivo.

### HOXA5 enhances the expression of MXD1 in ECCA cells

**Fig. 4:**
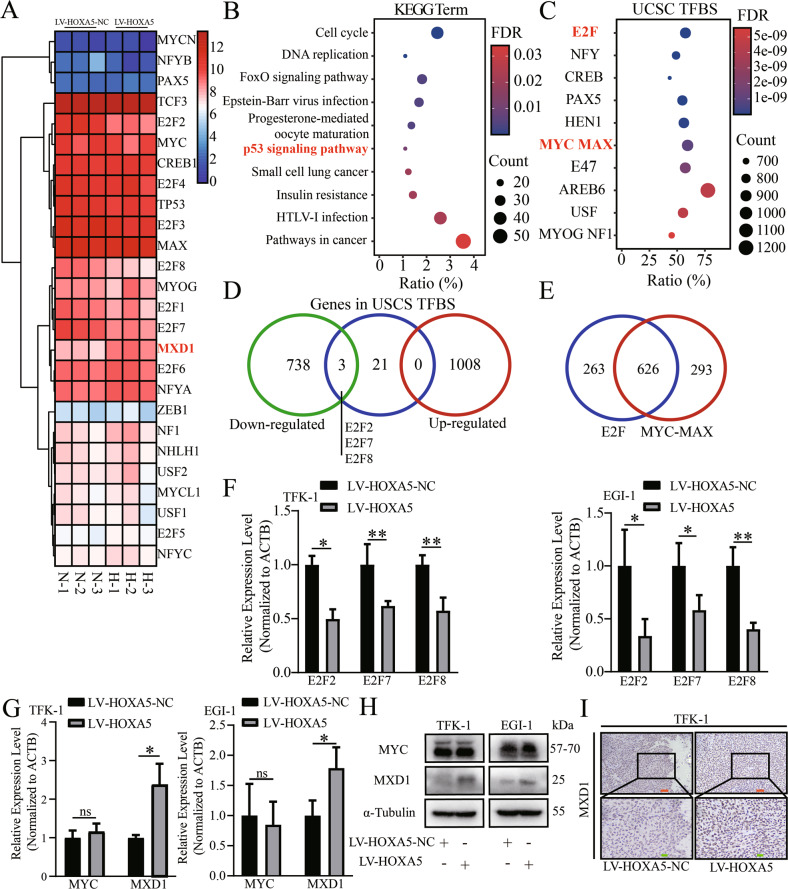
Identification of MXD1 as the potential target of HOXA5. **A** Heatmap analysis of the top DEGs from RNA-seq analysis of HOXA5 overexpression in ECCA cells. The gradual changes from blue to red indicate the expression levels from low to high. **B**, **C** Bubble plotted for the DEGs and TFBS enriched in the different pathways. The gradual changes from red to blue indicate in FDR from high to low. The sizes of bubbles represent the numbers of genes enriched in certain terms. **D** Venn diagram displayed the intersection between terms of TFBS and the DEGs. **E** Venn diagram exhibited the intersection between the predicted target gene of MYC-MAX and E2F terms. **F** Validation of E2F2, E2F7 and E2F8 expression levels by RT-qPCR in HOXA5 overexpressed cells. **G**, **H** Validation of MYC and MXD1 expression levels by RT-qPCR and Western blot in HOXA5 overexpressed cells. **I** Immunohistochemistry analysis of MXD1 expression in the tumors from nude mice. *P < 0.05. **P < 0.01. Orange bar, 50 μm. Green bar, 20 μm.

Next, we explored the potential targets of HOXA5 by RNA-seq analysis of LV-HOXA5 and LV-HOXA5-NC TFK-1 cells. Compared with the control cells, 1008 genes were up-regulated and 741 genes were down-regulated in LV-HOXA5 cells. Some DEGs are displayed in Fig. 4A. KEGG analysis indicated that the DEGs were enriched in the first three pathways of the “Cell cycle”, “DNA replication” and “FoxO signaling pathway” (Fig. 4B, Table S5), which regulated cell mitosis.

Due to the important role of HOXA5 in transcriptional regulation, we hypothesized that the downstream target of HOXA5 might be also a transcriptional regulator. Accordingly, we classified the DEGs, based on transcription factor binding site (TFBS). The first six TFs were “E2F”, “NFY”, “CREB”, “PAX5”, “HEN1” and “MYC MAX” (Fig. 4C, Table S6). We noticed the MYC-MAX transcription complex and its relevant first ten DEGs contained three genes in the E2F family (E2F2, E2F7, E2F8) (Fig. 4A, D), all of which are the downstream targets of MYC [24]. The E2F was predicted to target 889 genes while the MYC-MAX complex targeted 919 genes, which overlapped by nearly 70% of genes (Fig. 4E). Subsequently, RT-qPCR indicated that HOXA5 overexpression down-regulated E2F2, E2F7, and E2F8 mRNA transcripts in ECCA cells (Fig. 4F). Apparently, HOXA5 might regulate the transcription regulation ability of the MYC-MAX complex in ECCA cells.

MYC is one of the well-known oncogenes, contributing to the development and progression of cancer and functions through interaction with MAX [25]. We tested how HOXA5 could regulate the function of the MYC-MAX complex in ECCA cells. First, we found that HOXA5 overexpression failed significantly to alter the levels of MYC expression in ECCA cells (Fig. 4A, G, H). Intriguingly, HOXA5 overexpression significantly up-regulated the expression of MXD1, a tumor suppressor that can antagonize the formation of MYC-MAX complex [26], in ECCA cells (Fig. 4A, G, H). Further IHC confirmed an increase in MXD1 expression in HOXA5 over-expressed ECCA tumors from nude mice (Fig. 4I). These suggest that the MXD1 may be the downstream target of HOXA5 in ECCA cells.

### MXD1 acts as a tumor suppressor of cholangiocarcinoma

MXD1 is a tumor suppressor and functions to compete with MYC for interacting with MAX to form a transcription repressor of the MXD1-MAX [27]. Currently, little is known about the role of MXD1 in ECCA. To address it, we retrieved the expression profiles of MXD1 in Oncomine database. Among 37 results, 3 studies reported MXD1 up-regulation and 34 studies reported MXD1 down-regulation in cancerous tissues (Fig. S3H). The levels of MXD1 mRNA transcripts ranked the lowest in colorectal cancer (13 studies), leukemia (8 studies), esophageal cancer (5 studies) and head and neck cancer (4 studies) (Fig. S3I–N). Interestingly, the levels of HOXA5 mRNA transcripts were negatively correlated with MXD1 transcripts in GSE9348 (Fig. S3O), but positively with MXD1 in GSE13898 (Fig. S3P). To seek more evidence, we performed RT-qPCR to analyze the expression of MXD1 in human tissues. We found that MXD1 expression was down-regulated in ECCA tissues, compared with non-cancerous tissue, but there was no significant difference in the levels of MXD1 expression between ICCA and corresponding non tumor tissues (Fig. S5A, C). A similar pattern of positive correlation of HOXA5 and MXD1 transcripts was detected in ECCA tissues, but not in ICCA tissues (Fig. S5B, D). Accordingly, HOXA5 may regulate MXD1 expression in a tumor origination-dependent manner.

**Fig. 5:**
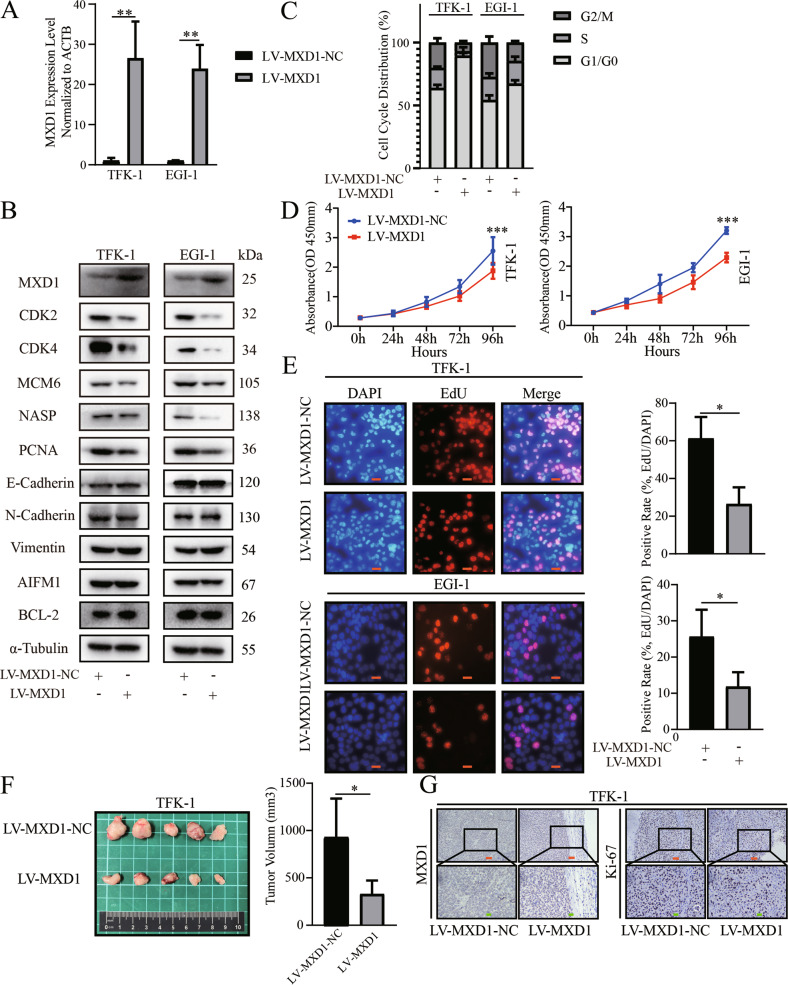
MXD1 overexpression reduces the malignant behaviors of ECCA cells. **A**, **B** RT-qPCR and Western blot validated MXD1 overexpression in ECCA cells. **B** Western blot analysis of the expression of mitosis-related genes in ECCA cells. MXD1 overexpression failed to modulate not only the EMT-related gene expression, but also the expression of AIFM1 and BCL-2 in ECCA cells. **C**–**E** MXD1 overexpression attenuated the cell cycle **C**, proliferation **D**, **E** of ECCA cells. **F** MXD1 overexpression decreased the tumor volumes in mice. **G** Immunohistochemistry analysis of MXD1 and Ki67 expression in the xenograft tumors from nude mice. Data are representative images of each group from three separate experiments. *P < 0.05. **P < 0.01. ***P < 0.001. Orange bar, 50 μm. Green bar, 20 μm.

Next, we evaluated the function of MXD1 in ECCA cells. We found that MXD1 overexpression obviously reduced the relative levels of CDK2, CDK4, MCM6, NASP and PCNA expression in ECCA cells (Fig. 5A, B). However, MXD1 overexpression did not significantly later the levels of HOXA5 expression in ECCA cells, indicating that there may not have a feedback loop between HOXA5 and MXD1 in transcription level (Fig. S5E, F). Functionally, MXD1 overexpression significantly attenuated the proliferation of ECCA cells, but failed to significantly alter the migration and spontaneous apoptosis in these cells (Fig. 5B–E, S6A, S6B). MXD1 overexpression induced cell cycling arrest in G0/G1 phase in both cell lines, similar to that of HOXA5 overexpression (Figs. 3C and 5C). MXD1 overexpression also inhibited the growth of implanted TFK-1 tumors by reducing their volumes and Ki67 expression (Fig. 5F, G). Collectively, these data imply that MXD1 may also be a tumor suppressor to inhibit the proliferation of ECCA cells.

### HOXA5 inhibits the proliferation of ECCA cells, dependent on up-regulating MXD1 expression

**Fig. 6:**
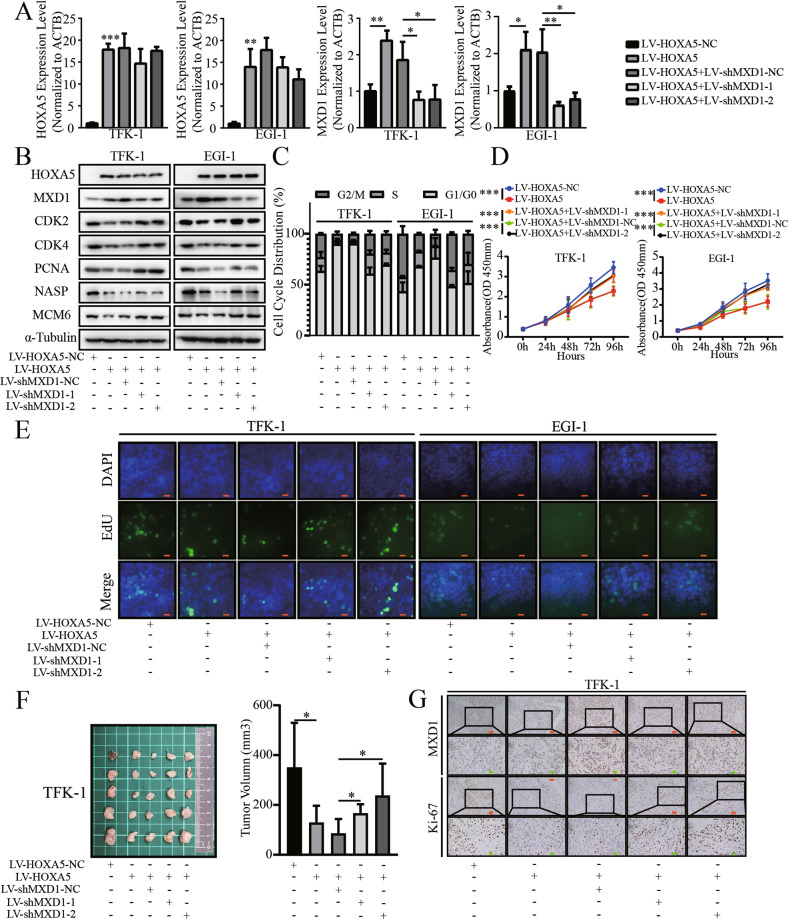
MXD1 silencing mitigates the inhibition of HOXA5 overexpression on the proliferation of ECCA cells. **A**, **B** MXD1 silencing did reduce MXD1, but not HOXA5 expression, and partially rescued the expression of mitosis-related genes in ECCA cells. **C**–**E** MXD1 silencing abrogated the inhibition of HOXA5 overexpression on the cell cycle and proliferation of ECCA cells. **F** MXD1 silencing partially rescued the tumor volumes in the HOXA5 over-expressing ECCA tumors from nude mice. **G** Immunohistochemistry analysis of MXD1 and Ki67 expression in xenograft tumors from nude mice. *P < 0.05. **P < 0.01. ***P < 0.001. Orange bar, 50 μm. Green bar, 20 μm.

To further explore whether HOXA5 inhibited the proliferation of ECCA cells, dependent on MXD1 up-regulation, we tested whether MXD1 silencing could mitigate and abrogate the reduced ECCA cell proliferation by HOXA5 overexpression. To address it, we transduced with lentivirus for the expression of MXD1-specific shRNAs in the HOXA5 over-expressed TFK-1 and EGI-1 cells (Fig. 6A, B). Functionally, we found that MXD1 silencing abrogated the reduced proliferation in the HOXA5 over-expressed TFK-1 and EGI-1 cells (Fig. 6B–E). Similarly, MXD1 silencing also partially or completely restored the levels of CDK2, CDK4, PCNA, NASP and MCM6 expression in the HOXA5 over-expressed TFK-1 and EGI-1 cells (Fig. 6B). In both cell lines, HOXA5 overexpression induced cell cycling arrest in G0/G1 phase, which were partially inhibited by MXD1 silencing (Fig. 6C). In addition, MXD1 silencing also restored the growth of implanted TFK-1 tumors and partially increased their levels of Ki67 expression in vivo, relative to that of HOXA5 over-expressing tumors (Fig. 6F, G). These data indicated that HOXA5 overexpression inhibited the proliferation of ECCA cells in a MXD1-dependent manner.

### HOXA5 directly induces the MXD1 transcription by binding to the MXD1 promoter

**Fig. 7:**
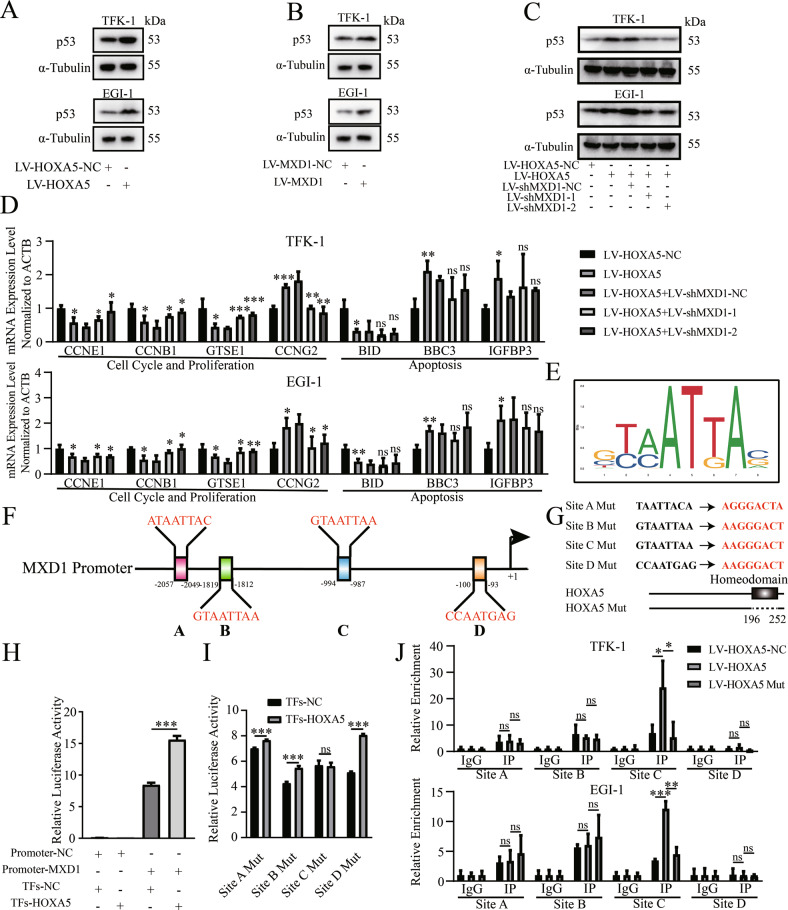
The HOXA5/MXD1 axis enhances p53 expression and HOXA5 directly induces MXD1 transcription in ECCA cells. **A**–**C** HOXA5 or MXD1 overexpression increased p53 protein expression, and the enhanced effect of HOXA5 overexpression on p53 protein expression was mitigated by MXD1 silencing in ECCA cells. **D** Validation of the target genes involved in the p53 pathway. **E** The predicted binding sequences of HOXA5 in the MXD1 promoter from the JASPAR database. **F** The predicted binding sites in the MXD1 promoter from the JASPAR database. **G** The sequences of four sites and the scheme of the mutations in this study. **H**, **I** Luciferase reporter assays indicated that HOXA5 expression enhanced the MXD1 promoter-controlled luciferase expression, which was abrogated by the site C mutation in the MXD1 promoter in HEK293T cells. **J** ChIP validated that HOXA5 expression increased its binding to the MXD1 promoter at the predicted site, which was inhibited by the mutation in the DNA binding domain of HOXA5. *P < 0.05. **P < 0.01. ***P < 0.001.

Finally, we tested whether HOXA5 could directly up-regulate MXD1 expression in ECCA cells. Actually, HOXA5 directly induces the expression of p53 in cervical cancer, breast cancer and MYC‑amplified medulloblastoma [4, 28, 29]. Intriguingly, we noticed that “the p53 signaling pathway” was enriched by HOXA5 overexpression in Fig. 4B and Table S5. Therefore, HOXA5 might interfere with the proliferation of cholangiocarcinoma cells by regulating the expression of p53, the hinge of p53 signaling pathway. We found that HOXA5 or MXD1 overexpression up-regulated p53 protein expression, but not mRNA transcripts in TFK-1 and EGI-1 cells (Fig. 7A, B, Fig. S7A). Furthermore, the enhanced p53 expression by HOXA5 overexpression was obviously abrogated by MXD1 silencing in TFK-1 and EGI-1 cells (Fig. 7C), indicating that HOXA5 up-regulated p53 expression, dependent on up-regulating MXD1 expression in ECCA cells.

To look for additional evidence, we analyzed 17 genes enriched in the “the p53 signaling pathway”. We found that 47.1% of them were involved in the regulation of cell cycle while 17.6% of them were associated with cell apoptosis (Fig. S7B). The expression of these genes is shown in Fig. S7C. We found that cell-cycle promoting factors, such as Cyclins and CDKs, were down-regulated while the cell cycling inhibitors, GADD45A and GADD45G, were up-regulated. Among the apoptosis-associated genes, some pro-apoptotic factors, such as BID, were not up-regulated, though HOXA5 overexpression could enhance spontaneous apoptosis (Fig. S4B). Further RT-qPCR revealed that three cell-cycle promoting factors (CCNE1, CCNB1 and GTSE1) were down-regulated in the HOXA5 overexpressed cell, which were rescued by MXD1 silencing (Fig. 7D). In contrast, pro-apoptotic factors were up-regulated in the HOXA5 over-expressed cells, except for BID. However, their gene transcripts were not significantly altered by MXD1 silencing, consistent with the finding described above (Fig. 7D, Fig. S6B). Besides, the expression of CCNG2, a cell cycling inhibitor involved in negative feedback of p53, was up-regulated, which could be regarded as a compensatory reaction to p53 activation (Fig. 7D).

To learn the binding pattern of HOXA5 in the genome, we performed CUT & Tag analysis. A total of 7073 peaks, involved in different binding sites of 4579 genes, were found in Flag-HOXA5 over-expressed TFK-1 cells. The distribution of these peaks is shown in Fig. S8A, B. Most peaks were situated in the promoter region (37.78%) and distal intergenic regions (34.71%). In the promoter region, most binding sites (33.11% sites in the region within 1000 bps, 2.51% sites between 1000 and 2000 bps, 2.16% sites between 2000 and 3000 bps) were located within 1000 bps from the transcription initiation site (TSS) (Fig. S8C). Analysis of the RNA-seq and CUT & Tag data together revealed that 353 DEGs were likely regulated by HOXA5 directly (Fig. S8D). The GO analysis indicated that some DEGs were involved in cell proliferation, such as “DNA replication” and “Regulation of cell cycle”, paralleling with the findings that HOXA5 inhibited the proliferation of ECCA cells (Fig. 3B–E and Fig. S8E, Table S7). Interestingly, 1 peak in the MXD1 promoter region was further analyzed (Fig. S8F).

**Fig. 8:**
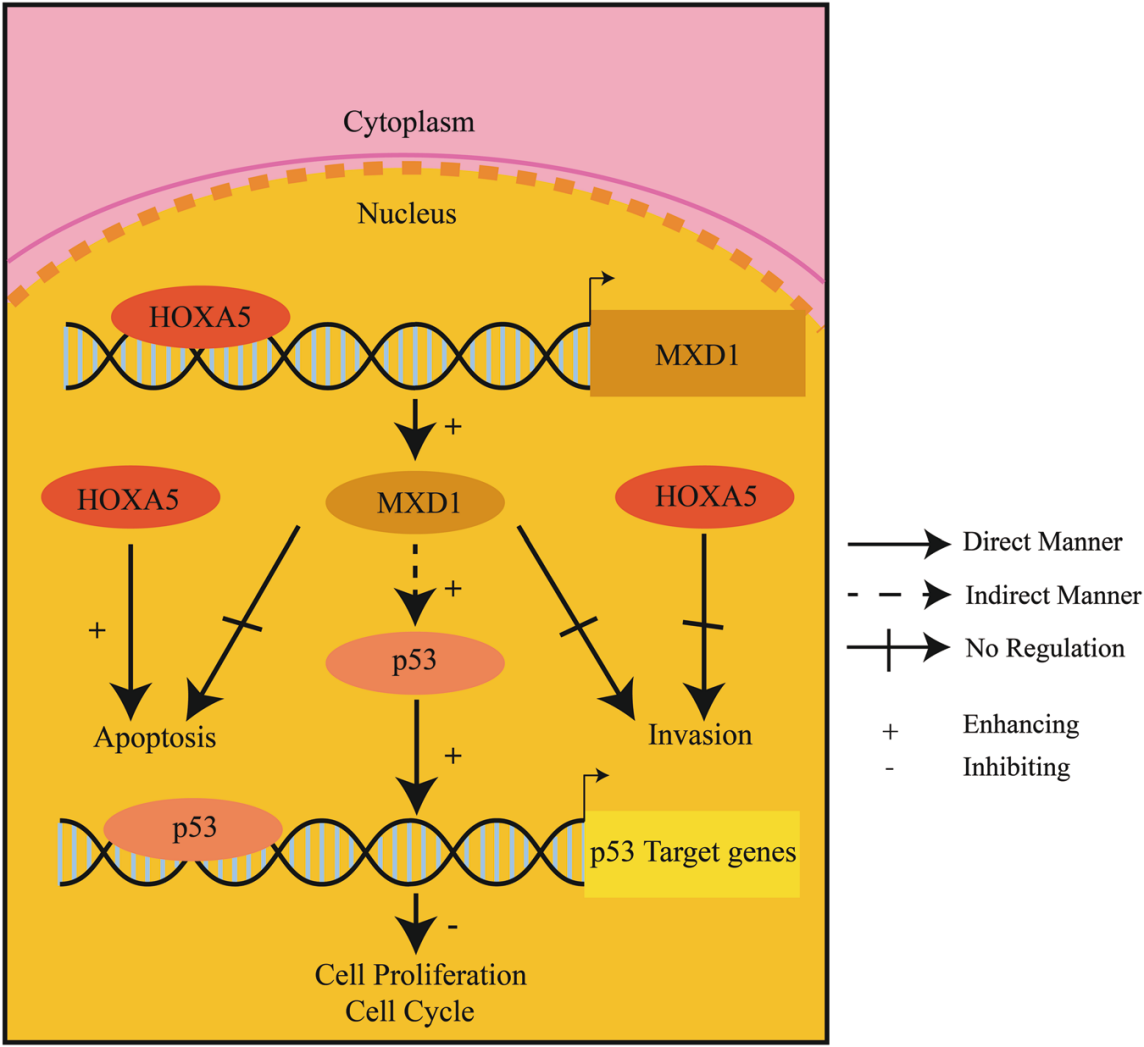
A diagram illustrates the roles of HOXA5 and MXD1 in the pathogenesis of ECCA. HOXA_5_ directly induced the expression of MXD_1_ by binding to the MXD_1_ promoter region. The up-regulated MXD1 expression indirectly enhanced the p53 signaling to inhibit the proliferation of ECCA cells. HOXA5 could induce the apoptosis of cholangiocarcinoma cells, but have no significant effect on their invasion ability.

Subsequently, we tested whether HOXA5 could directly up-regulate MXD1 expression in ECCA cells. First, we selected the matrix profile MA0158.2 for further analysis (Fig. 7E). According to the JASPAR database, we predicted the binding sites of HOXA5 in the MXD1 promoter and selected the first four positive strand sequences, based on their scores (Fig. 7F). The sequences of these four sites are shown in Fig. 7G. Luciferase reporter assays revealed that co-transfection with plasmids for HOXA5 expression and the MXD1 promoter-controlled luciferase expression significantly enhanced the MXD1 promoter-controlled luciferase expression in HEK293T cells (Fig. 7H). Further luciferase reporter assays displayed that co-transfection with the plasmids for HOXA5 expression and a site-specific mutant at the MXD1 promoter Site C, but not the sites of A, B, and D, abrogated the HOXA5-enhanced luciferase activity in HEK293T cells, as compared with that of transfection with wild-type of promoter in our experimental condition (Fig. 7I). Interestingly, the site C was close to the peak we found in the MXD1 promoter region. The scheme for the site-directed mutation of four sites is shown in Fig. 7G. These suggest that HOXA5 may bind to the MXD1 promoter at Site C to induce MXD1 expression. Finally, ChIP assays exhibited that anti-Flag antibody significantly precipitated the MXD1 promoter region containing the site C, but not other three sites tested, in both TFK-1 and EGI-1 cells compared with the IgG control (Fig. 7J). The binding could be inhibited by deletion of Homeodomain, the DNA binding domain of HOXA5 (Fig. 7G, J). Therefore, HOXA5 is directly bound to the MXD1 promoter at the site of -994 to -987 to induce MXD1 expression, activating the p53 pathway to inhibit the proliferation of ECCA cells (Fig. 8).

## Discussion

The gene methylation is critical for its expression and can regulate the development and progression of malignant tumors [20]. In the present study, we found hypermethylation in the promoters of many HOX genes, particularly for *HOXA5*, associated with down-regulated HOXA5 expression in cholangiocarcinoma and ECCA cells. The down-regulated HOXA5 expression was restored by DCA treatment in ECCA cells and associated significantly with worse OS of ECCA, but not ICCA, patients. Apparently, the down-regulated HOXA5 expression contributed to the development and progression of ECCA.

Next, we investigated the function of HOXA5 in the malignant behaviors of ECCA cells. We found that HOXA5 overexpression inhibited the proliferation of ECCA cells by enhancing their apoptosis, but did not significantly modulate their migration. HOXA5 overexpression also inhibited the growth of implanted ECCA tumors in vivo and down-regulated the mitosis and survival-related, but not the EMT-related gene expression in ECCA cells. These data extended previous observations in other types of cancers [4, 7, 30] and support the notion that HOXA5 acts as a tumor suppressor to inhibit the progression of ECCA.

Given that HOXA5 is a transcription factor we explored the potential targets of HOXA5 in ECCA cells by RNA-seq and CUT & Tag. We found that HOXA5 overexpression modulated many gene expressions, including up-regulated MXD1 expression in ECCA cells. The DEGs were enriched in several pathways that regulated the progression of cancer. The MYC-MXD1 signaling is crucial for tumorigenesis and cancer progression [13, 25]. We found that HOXA5 did not significantly change MYC expression levels, but significantly up-regulated MXD1 expression and down-regulated E2F2/7/8 expression in ECCA cells. Functionally, MXD1 overexpression also inhibited the proliferation and tumor growth of ECCA cells, but did not significantly alter their migration and apoptosis. The result of apoptosis analysis upon MXD1 overexpression was found conflicting with the anti-tumor effects of HOXA5. The reason might be that HOXA5 induced the apoptosis by activating other pathways, independent of the function of MXD1 because MXD1 silencing failed to significantly alter the expression levels of apoptosis-related genes in ECCA cells. More importantly, MXD1 silencing abrogated the HOXA5-decreased proliferation and tumor growth of ECCA cells. HOXA5 is directly bound to the MXD1 promoter to enhance the MXD1-controlled luciferase expression in HEK-293T cells. Such novel data indicated that HOXA5 directly induced MXD1 expression, and inhibited the mitosis of ECCA cells in a MXD1-dependent manner.

Constitutive activation of the p53 signaling inhibits cancer growth [31]. HOXA5 directly induces p53 expression in cervical cancer, breast cancer, and MYC‑amplified medulloblastoma [4, 28, 29]. In this study, we found that not only HOXA5, but also MXD1 overexpression increased p53 protein, but did not significantly alter the p53 mRNA transcripts in ECCA cells. Furthermore, the enhanced p53 protein by HOXA5 overexpression was abrogated by MXD1 silencing in ECCA cells. Hence, HOXA5 increased p53 in a MXD1-dependent manner. Given that HOXA5 or MXD1 overexpression did not alter p53 mRNA transcripts our data suggest that the increased p53 protein by HOXA5 or MXD1 overexpression may be regulated by their related signaling to enhance p53 protein translation or inhibit p53 protein degradation. These data also suggest that regulation of the p53 signaling by HOXA5 and MXD1 may vary in different types of cancers. We are interested in further investigating how the HOXA5/MXD1 signaling regulates p53 expression during the process of ECCA. Nevertheless, our findings indicated that HOXA5 acted as a tumor suppressor to up-regulate MXD1 expression, enhancing the p53 signaling to inhibit the proliferation of ECCA cells. Thus, the HOXA5/MXD1 axis may be a potential therapeutic target for ECCA.

We recognized that our study had limitations. First, the sample size was relatively small due to low incidence and surgical rates of ECCA. Second, we have no data about how the HOXA5/MXD1 axis regulates p53 expression in ECCA because HOXA5 increased p53 protein in ECCA cells in a MXD1-dependent manner. Given that MXD1 interaction with MAX to form a transcription repressor to inhibit the activity of their regulated transcription factors and HOXA5 did not up-regulate p53 transcription the HOXA5/MXD1 may enhance p53 translation and/or attenuate its degradation in ECCA cells. These should be attributed to complex regulation and need to be investigated in the future.

## Conclusions

In summary, our data indicated that hypermethylation in the HOXA5 promoter down-regulated its expression in ECCA and was associated with worse OS in ECCA patients. Functionally, HOXA5 overexpression inhibited the proliferation and growth of ECCA in a MXD1-dependent manner and HOXA5 was directly bound to the MXD1 promoter to induce its expression. Furthermore, HOXA5 overexpression increased p53 protein in ECCA cells, dependent on up-regulating MXD1 expression. Our novel data indicated that HOXA5 acted as a tumor suppressor to inhibit the growth of ECCA by up-regulating MXD1 and p53 expression and HOXA5/MXD1 may be new therapeutic targets for intervention of ECCA. Hence, our findings may uncover molecular mechanisms by which the HOXA5/MXD1 signaling regulates the malignant behaviors of ECCA.

## Availability of data and materials

The datasets downloaded from GEO database and analyzed during the present study are available from the corresponding author upon reasonable request. The microarray and high throughput sequencing data were uploaded to GEO database (GSE211907, GSE211911, and GSE212058).

## Supplementary information

Supplementary Figures_Sep16

Table S1

Table S2

Table S3

Table S4

Table S5

Table S6

Table S7

Western_Aug23

aj-checklist
