# Supplementary material for: HOXA5 inhibits the proliferation of extrahepatic cholangiocarcinoma cells by enhancing MXD1 expression and activating the p53 pathway
Source: Cell Death Dis. 2022 Sep 27;13(9):829. doi: 10.1038/s41419-022-05279-6 (PMC9515223; doi:10.1038/s41419-022-05279-6)

**Figure S1.** Analysis of the expression of hypermethylated HOX genes mentioned in Figure 1. A. Heatmap analysis of the expression levels of the HOX genes in bile duct, pancreas and liver tissues, based on GEPIA database. The gradual changes from black to white represent changes in expression levels from high to low. B. RT-qPCR analysis of the expression levels of these HOX genes following DCA treatments in ECCA cells.

**
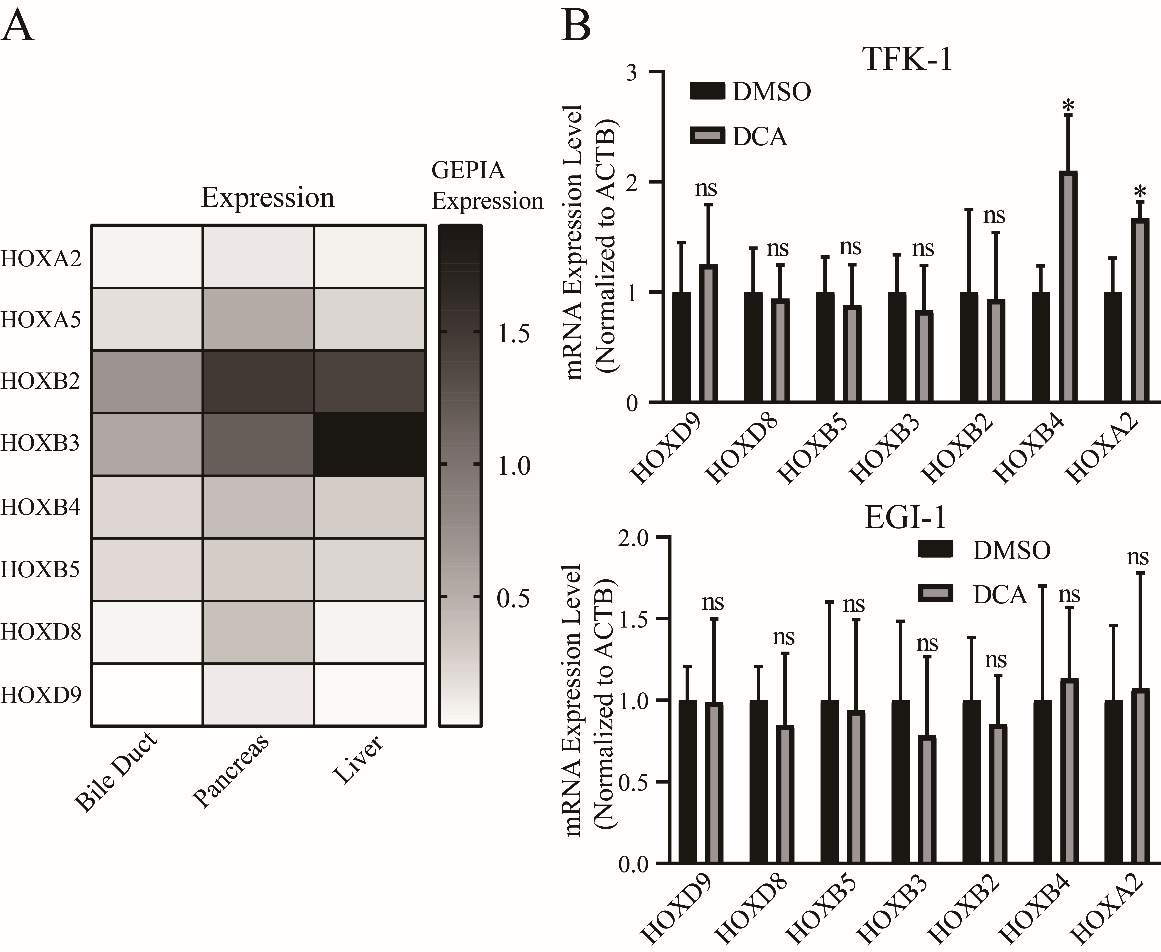
**

**Figure S2.** HOXA5 expression in ICCA tissues. A. Immunohistochemistry analysis of HOXA5 expression in ICCA and non-tumor tissues. B. RT-qPCR analysis of HOXA5 expression in ICCA and paired non-tumor tissues. C. The association between HOXA5 expression and the survival of ICCA patients. HOXA5 was not associated significantly with the survival of ICCA patients. Red bar, 100 μm. Black bar, 40 μm.

**
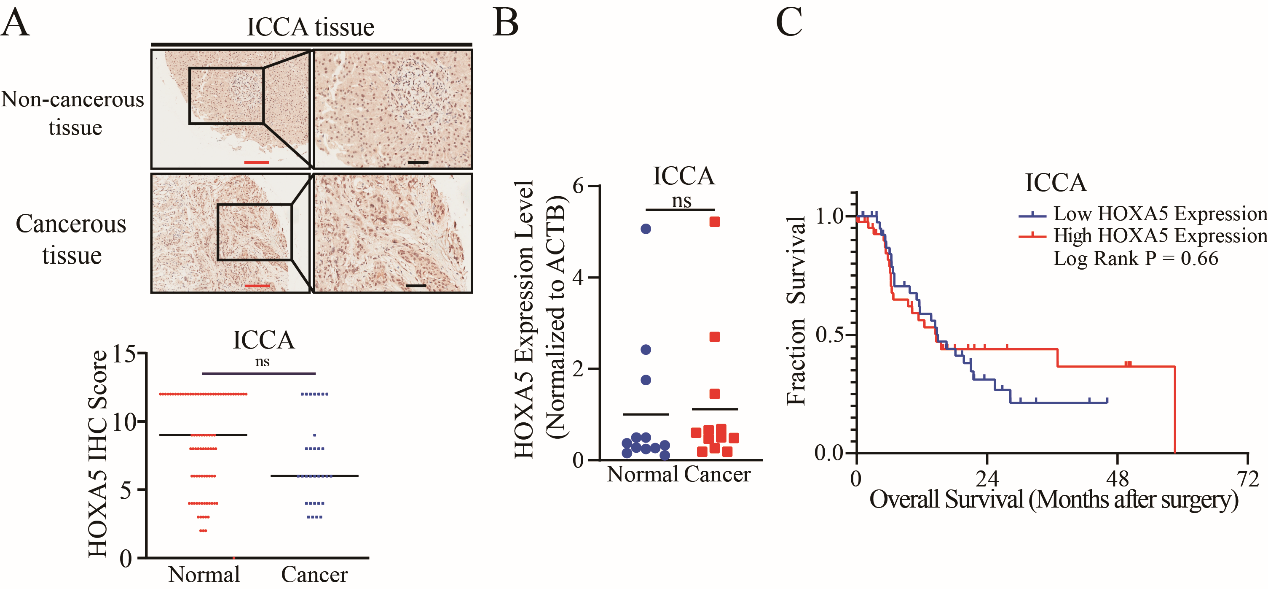
**

**Figure S3.** The HOXA5 and MXD1 transcripts between cancerous and para-tumor tissues in Oncomine and GEO database. A, H. The number of datasets with up-regulated HOXA5 and MXD1 mRNA transcripts (red) or downregulated transcripts (blue) in Oncomine database. The threshold was designed with the following parameters: P value of 1E^−4^ and fold change of 2. B-G. The expression profiles of HOXA5 in GEO database. I-N. The expression profiles of MXD1 in GEO database. O, P. Analysis of the correlation between HOXA5 and MXD1 expression in GSE9348 and GSE13898 datasets. **P*<0.05. ****P*<0.001.


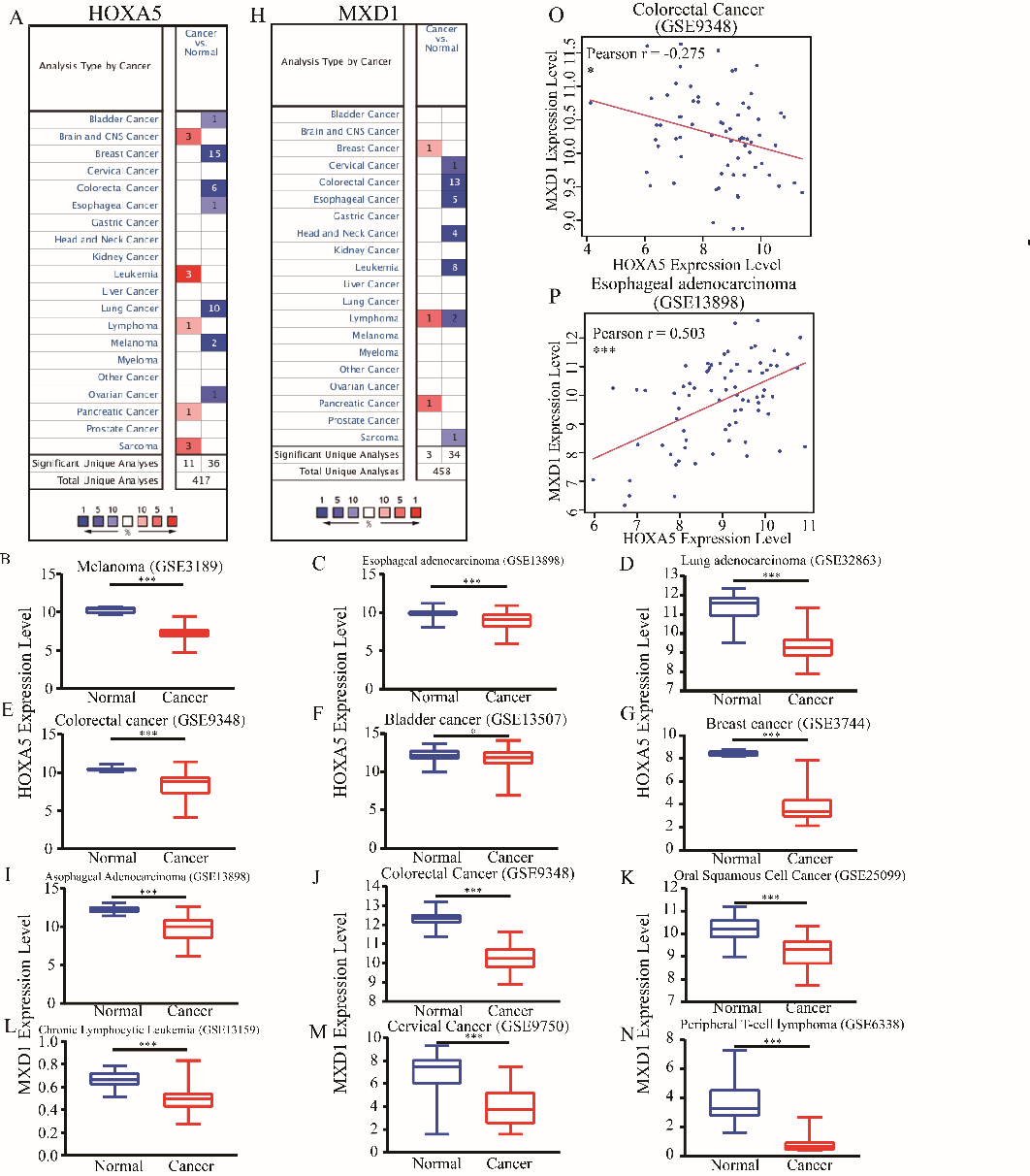


**Figure S4.** Measurement of the invasion ability and apoptosis of cholangiocarcinoma cells after inducing HOXA5 over-expression. A. HOXA5 over-expression did not alter the wound healing of ECCA cells. B. HOXA5 over-expression increased the frequency of apoptotic cells. **P*<0.05. ***P*<0.01.


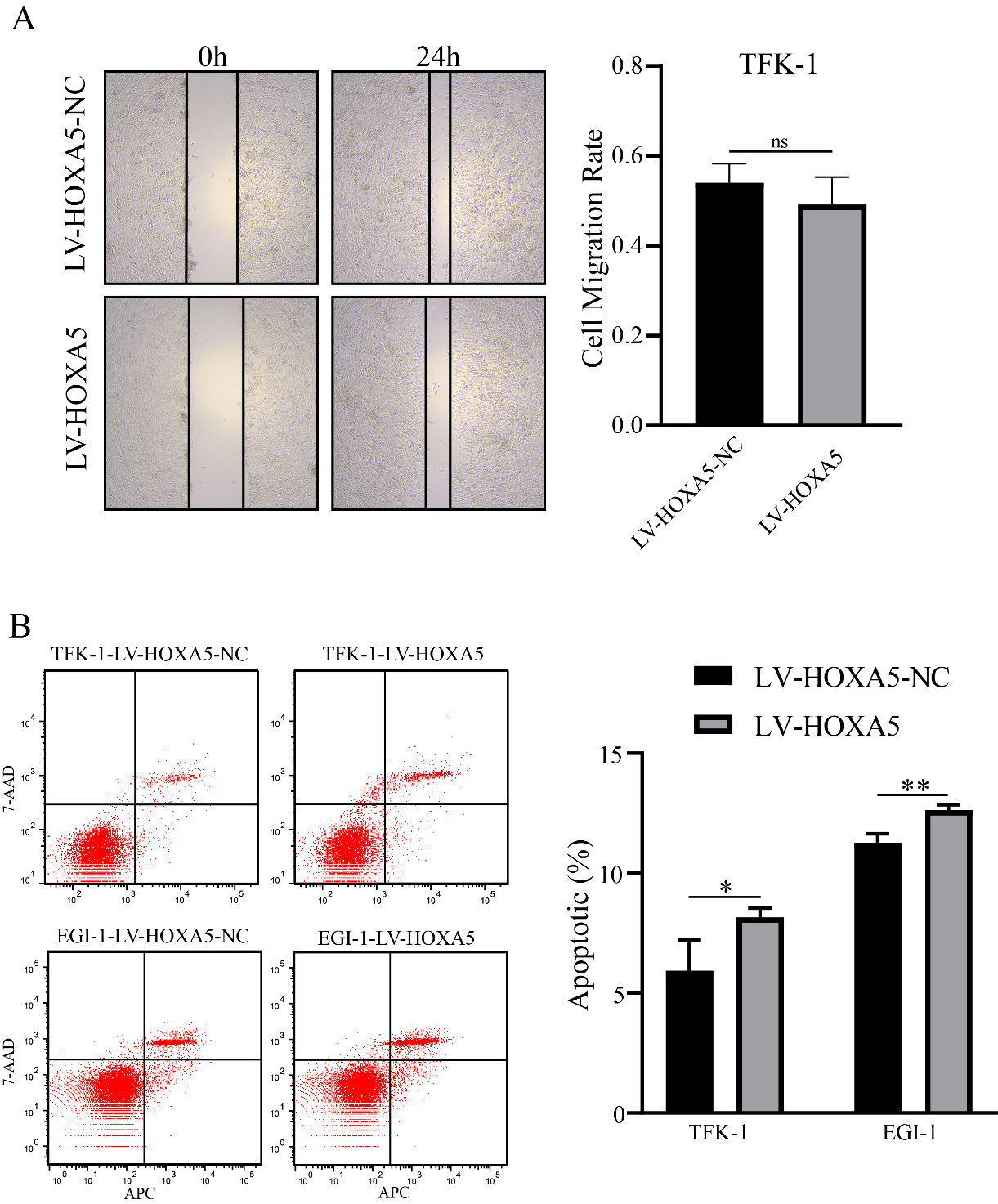


**Figure S5.** Evaluation of the MXD1 expression in human tissues and the effect of MXD1 over-expression on HOXA5 expression. A, B. MXD1 was up-regulated in ECCA tissues compared with paired non-cancerous tissues. The expression of MXD1 was positively-correlated with that of HOXA5 in ECCA tissues. C, D. There was no significant difference in the levels of MXD1 expression between ICCA and paired non-cancerous tissues. There was no significant correlation between HOXA5 and MXD1 expression. E, F. RT-qPCR and Western blot revealed that MXD1 over-expression did not significantly alter the levels of HOXA5 expression in ECCA cells. **P*<0.05.

**
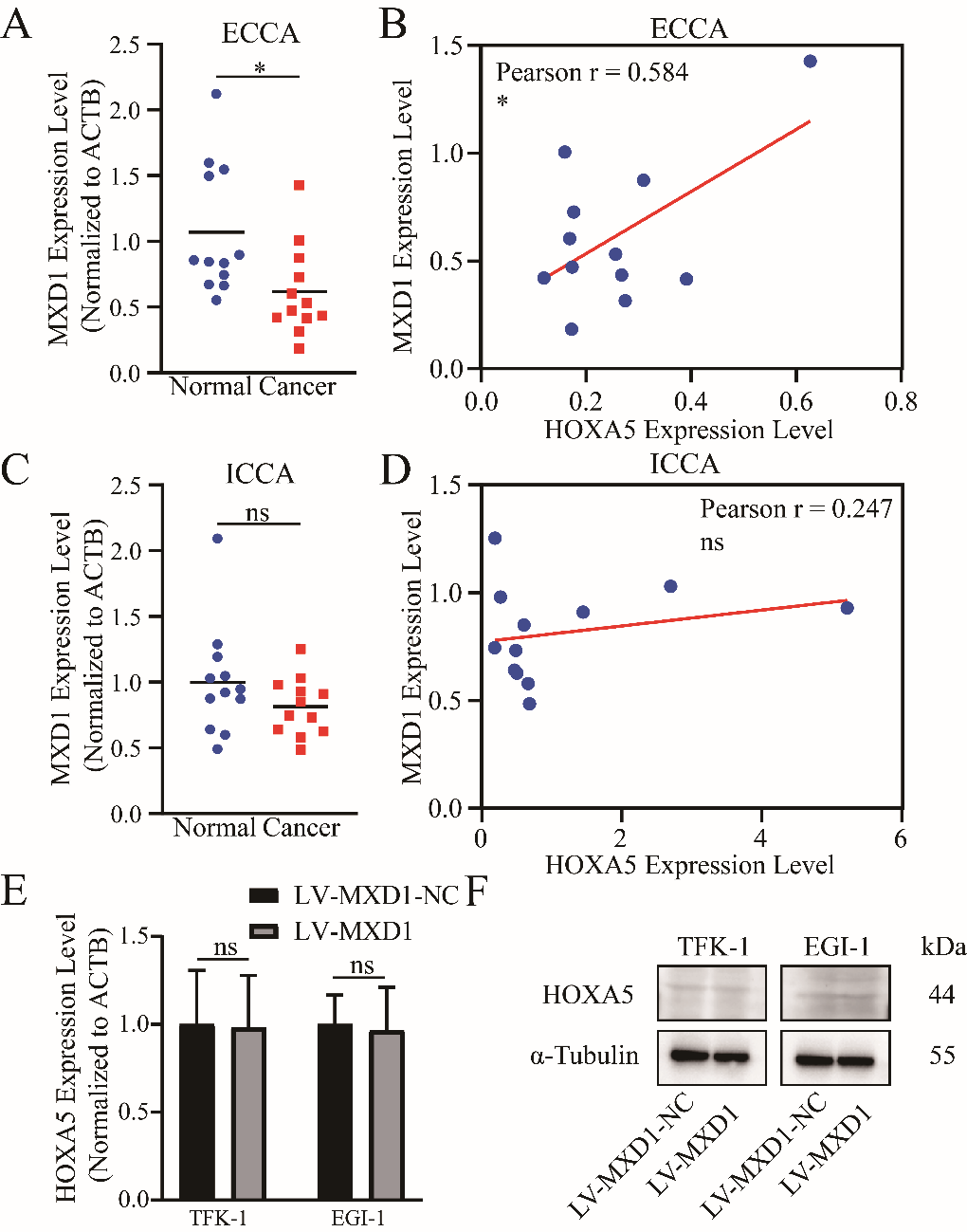
**

**Figure S6.** Measurement of the migration ability and apoptosis of cholangiocarcinoma cells upon HOXA5 over-expression. A. MXD1 over-expression did not alter the wound healing of ECCA cells. B. Flow cytometry analysis of the frequency of apoptotic cells.


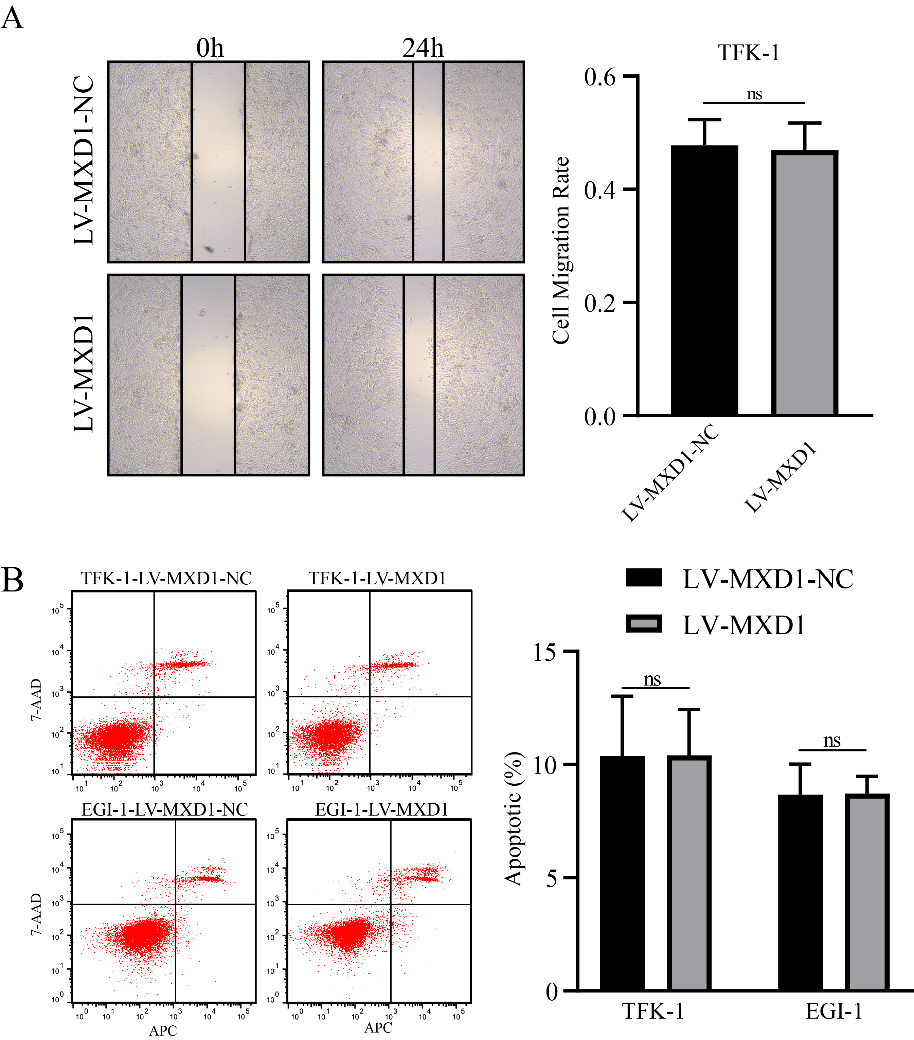


**Figure S7.** A summary of the genes involved in the p53 pathway. A. HOXA5 or MXD1 over-expression did not significantly change the relative levels of p53 mRNA transcripts in ECCA cells. B. The function of these genes and the proportion of each term. C. The genes in the signal flow diagram downloaded from KEGG. Red boxes represent up-regulated genes. Blue boxes represent down-regulated genes.


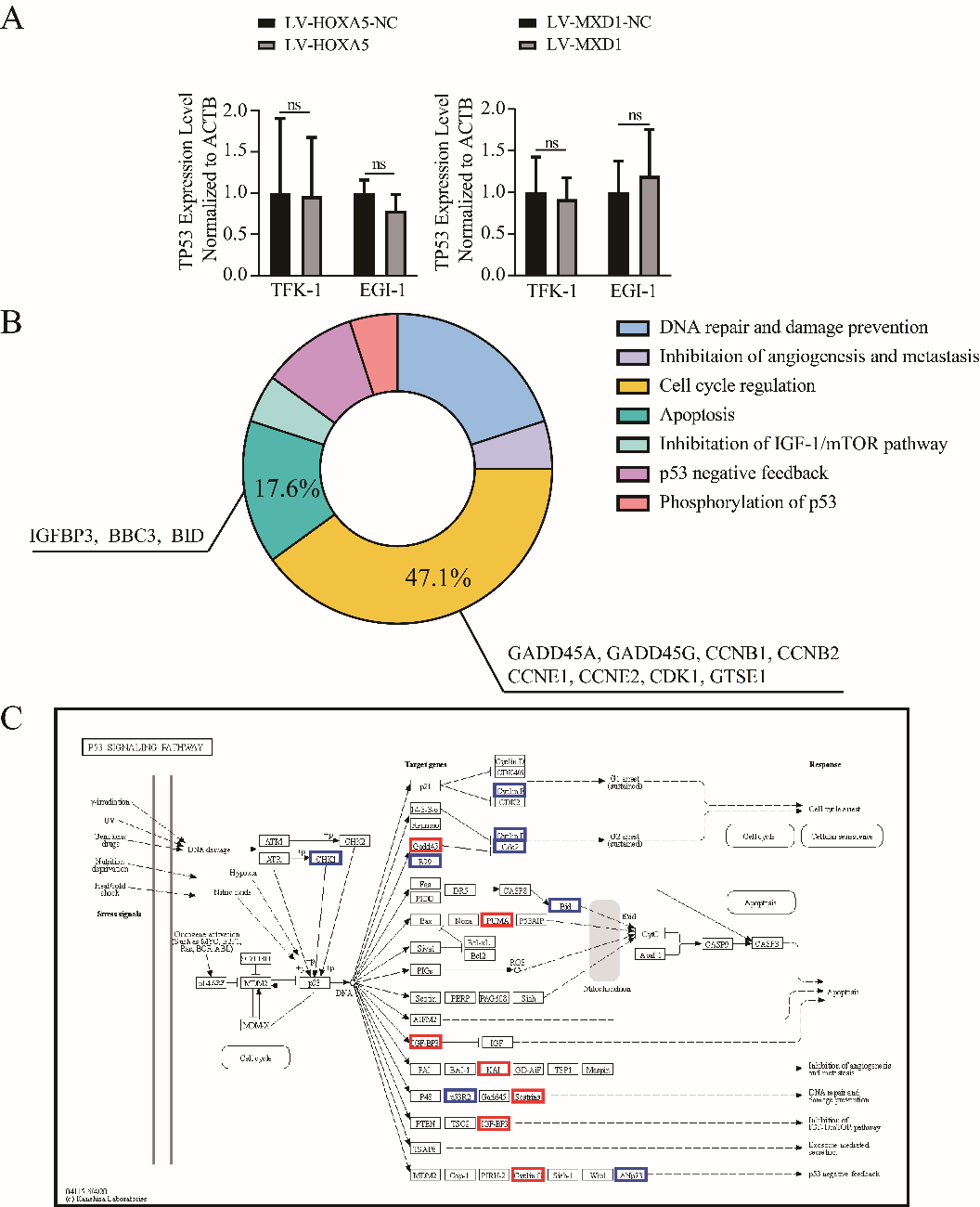


**Figure S8.** The CUT & Tag assay reveals the binding pattern of HOXA5 in the genome of ECCA cells. A. The distribution of peaks in the genome of HOXA5 expressed cells. B. The distribution of peaks around the TSS sites of different genes. C. A summary of the situations of the peaks in different regions. D. Calculation of the intersection between genes containing at least 1 peak and DEGs in RNA-seq. E. GO analysis of the genes in the intersection. The gradual changes from red to blue indicate in FDR from high to low. The sizes of bubbles represent the numbers of genes enriched in certain terms. F. A peak in the promoter region of the MXD1 gene.


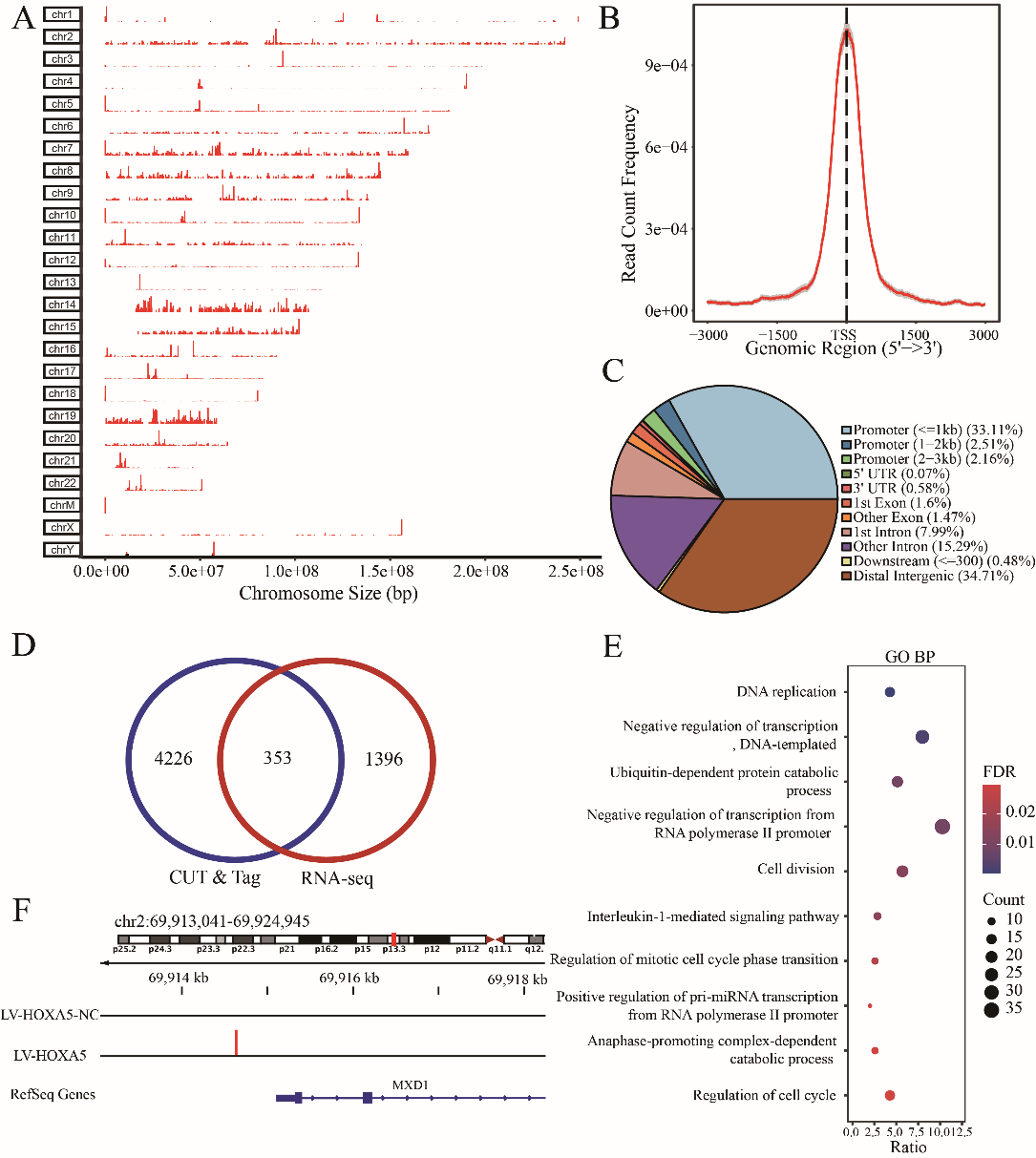

Supplement: Supplementary file 1 — Supplementary Figures_Sep16 [file 41419_2022_5279_MOESM1_ESM.docx]
