# Supplementary material for: HOXA5 inhibits the proliferation of extrahepatic cholangiocarcinoma cells by enhancing MXD1 expression and activating the p53 pathway
Source: Cell Death Dis. 2022 Sep 27;13(9):829. doi: 10.1038/s41419-022-05279-6 (PMC9515223; doi:10.1038/s41419-022-05279-6)
Supplement: Supplementary file 9 — Western_Aug23 [file 41419_2022_5279_MOESM9_ESM.docx]

Figure 1

Figure 1H


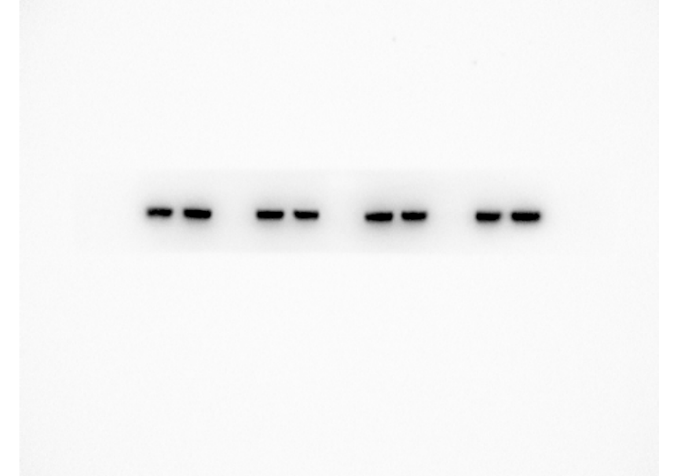

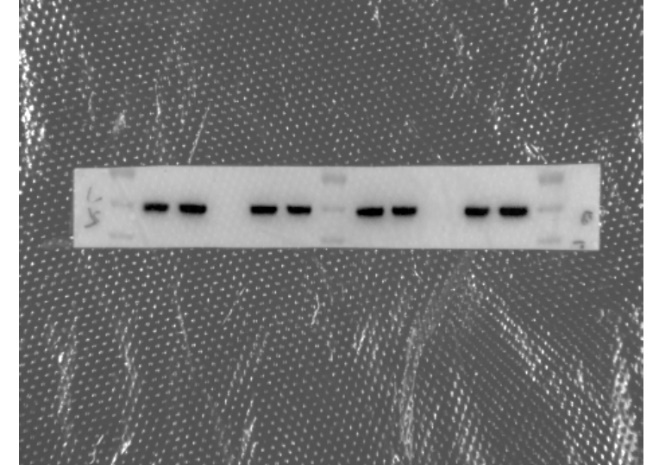
α-Tubulin


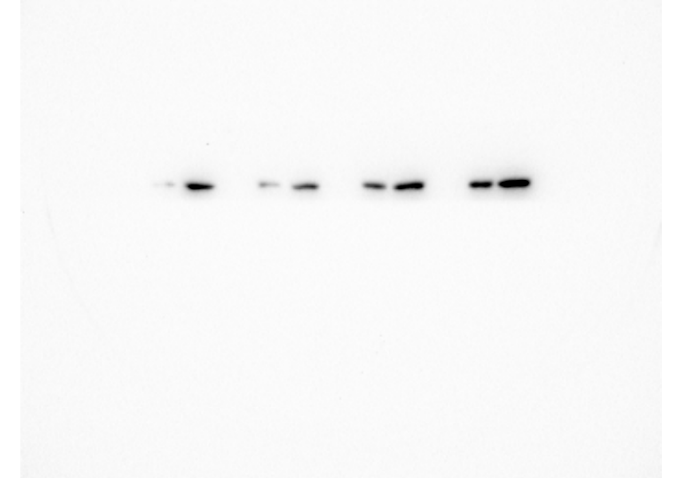

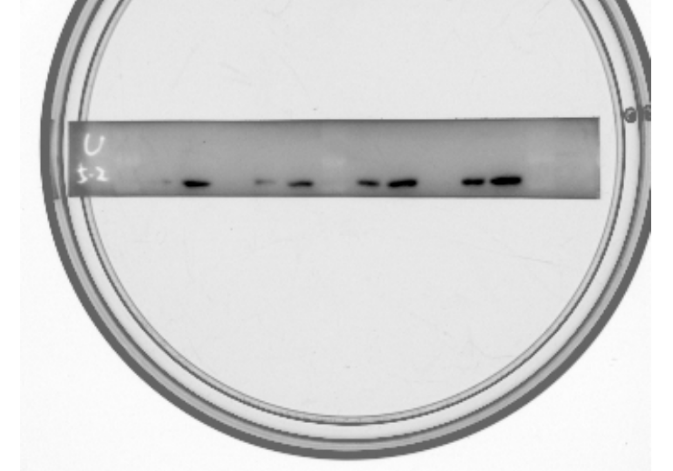
HOXA5

Figure 2


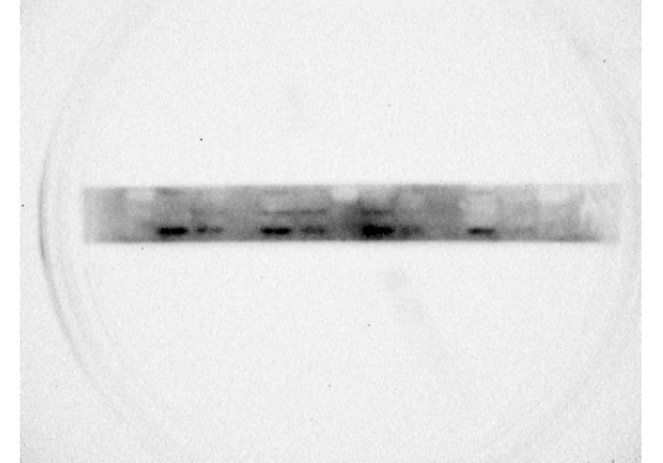
Figure 2F


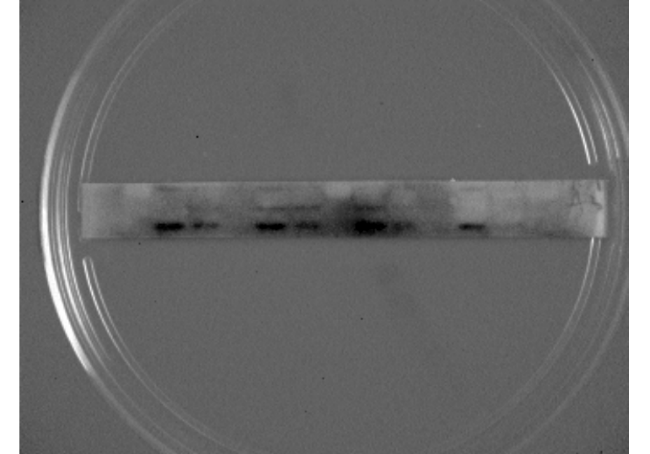
HOXA5 (1-3)


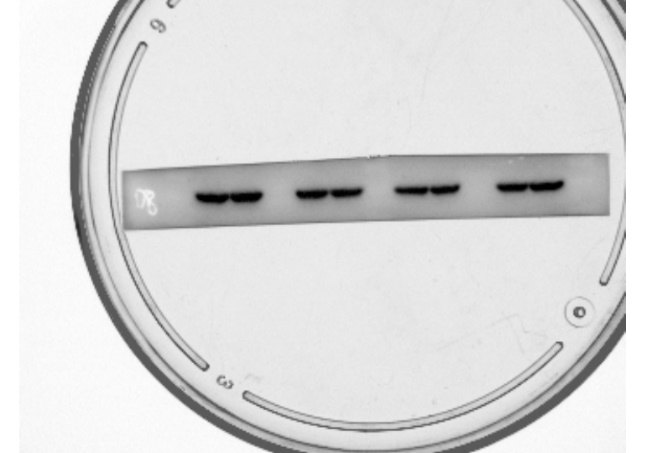

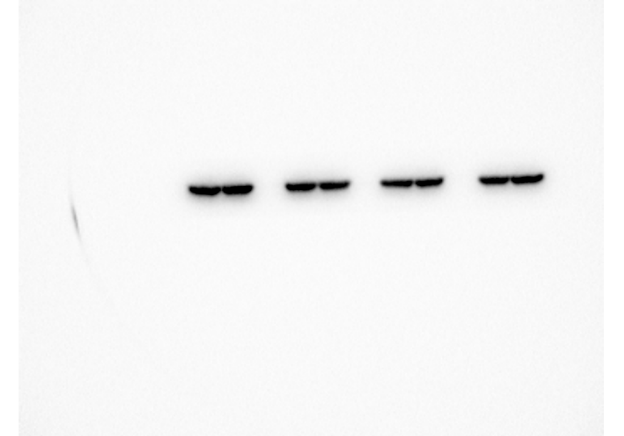
α-Tubulin (1-3)


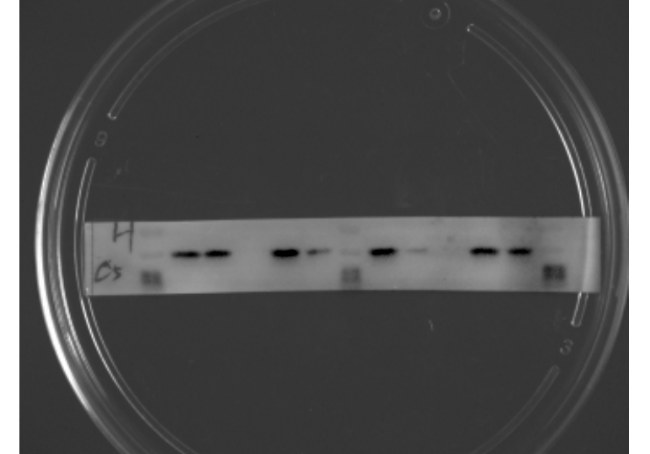

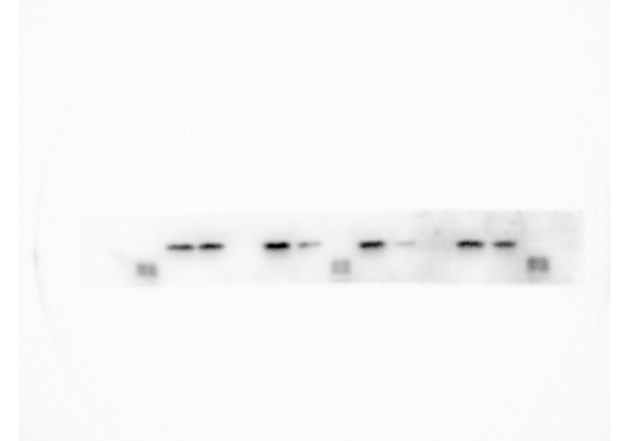
HOXA5 (4, 5, 11)


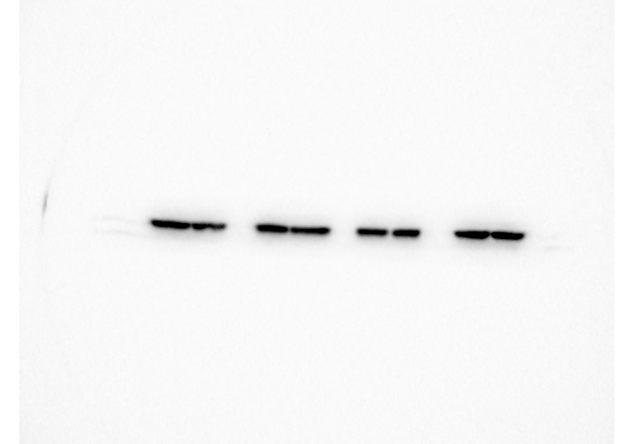

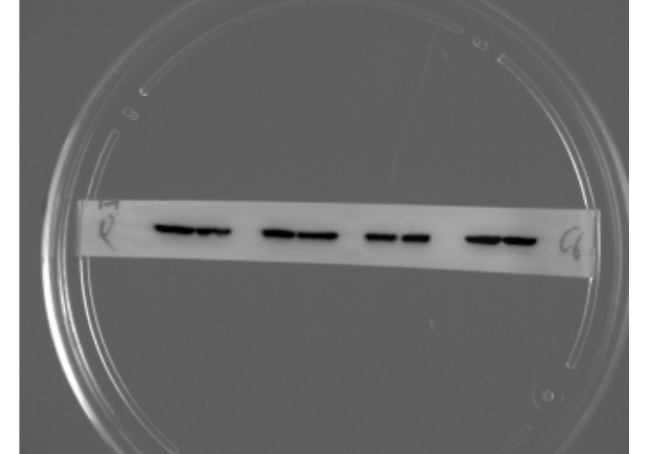
α-Tubulin (4, 5, 11)


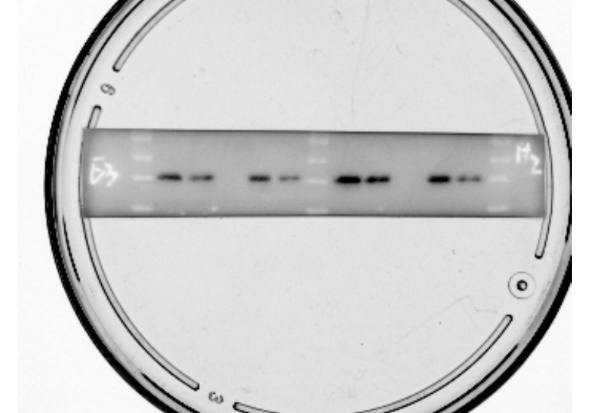

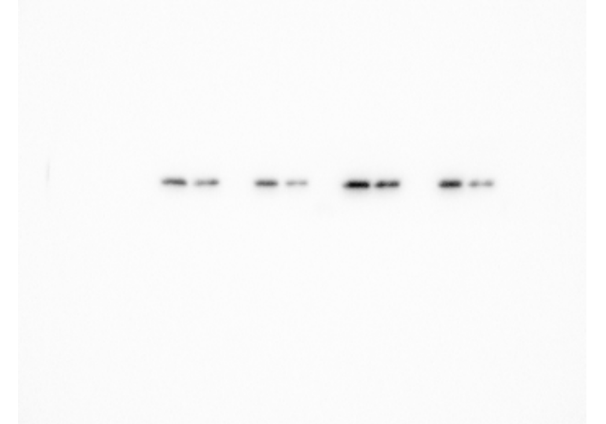
HOXA5 (6-8)


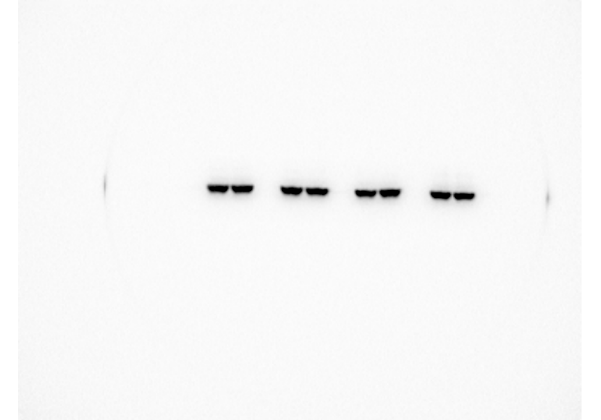

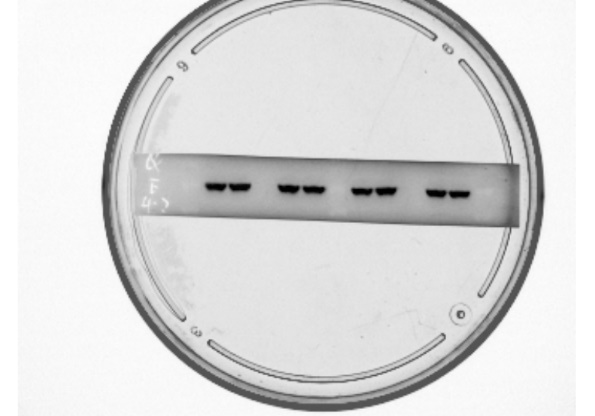

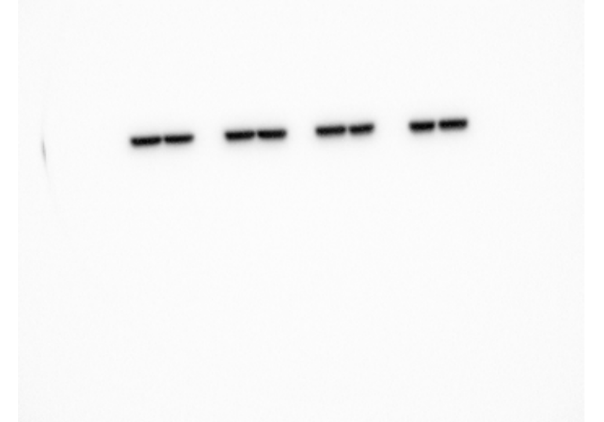

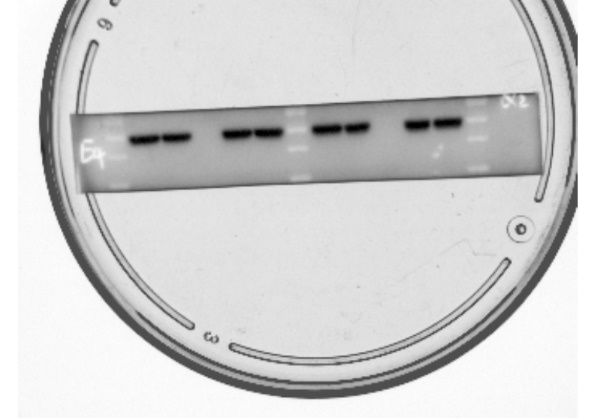
α-Tubulin


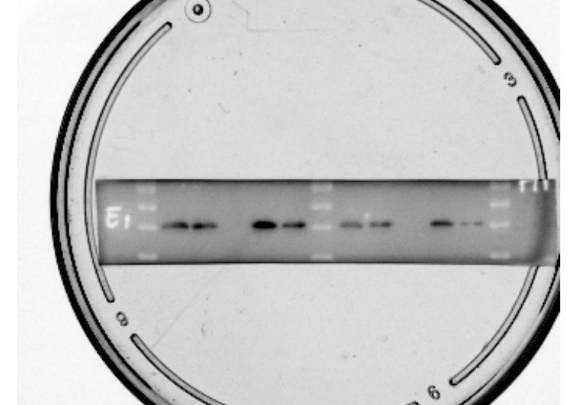
HOXA5 (9, 10, 12)


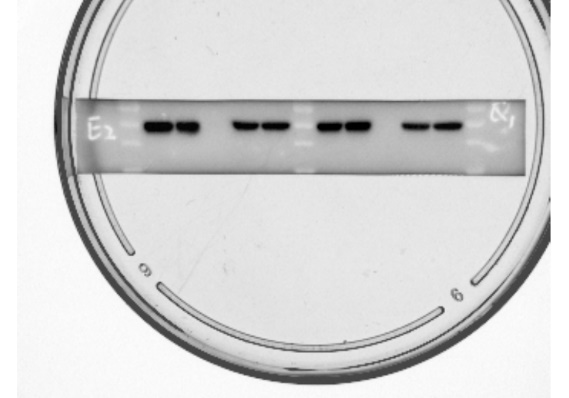
α
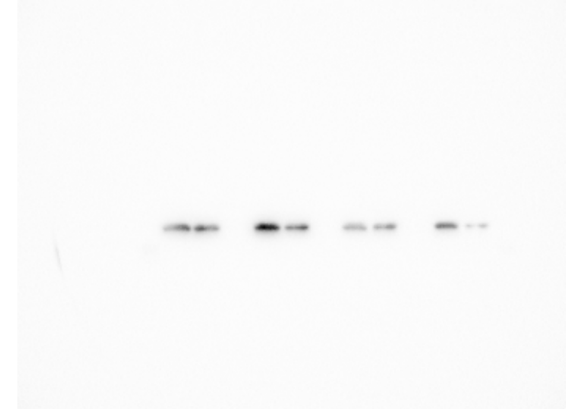
-Tubulin (9,10,12)

Figure
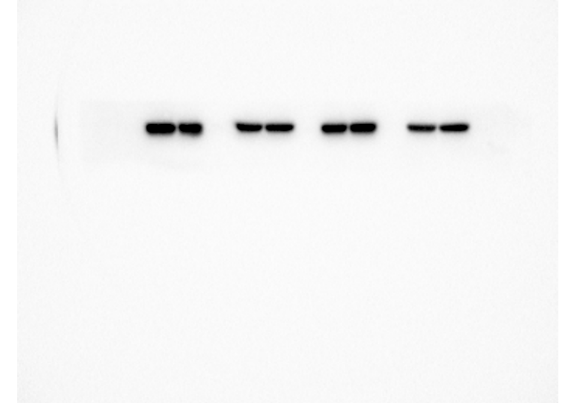
 3

Figure 3B


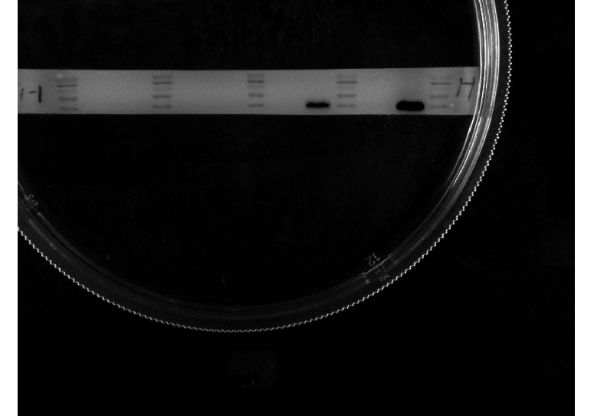

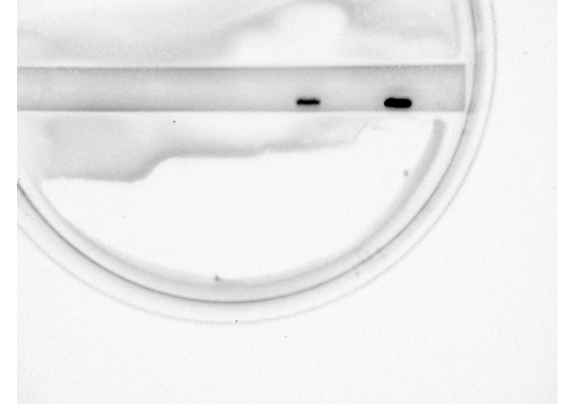
HOXA5 (TFK-1)


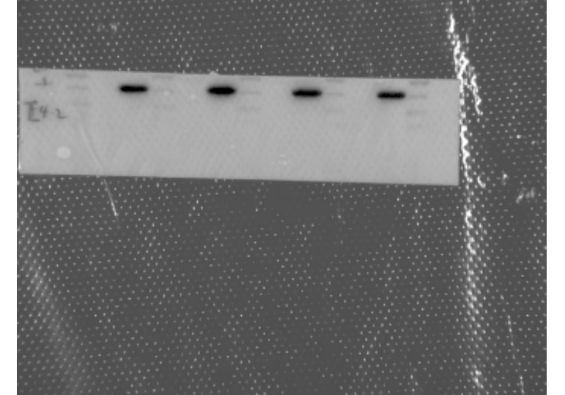

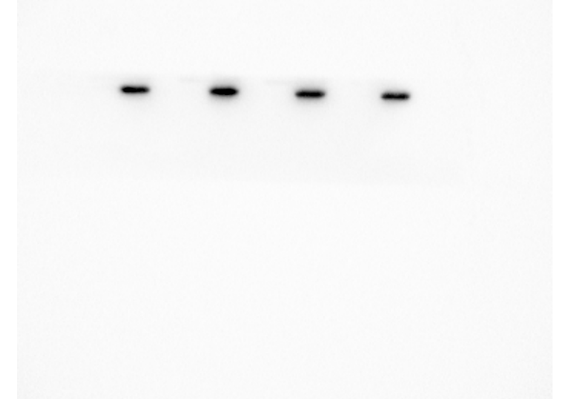
HOXA5 (EGI-1)


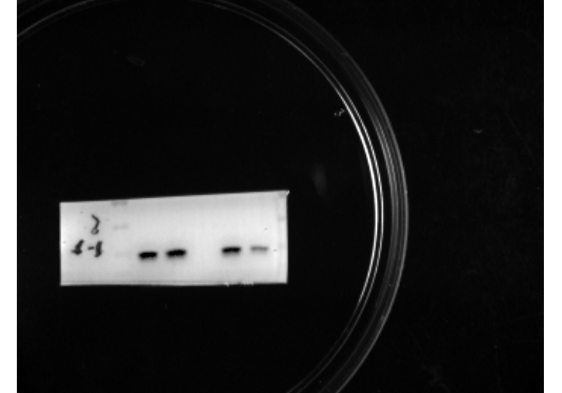
CDK2 (TFK-1)


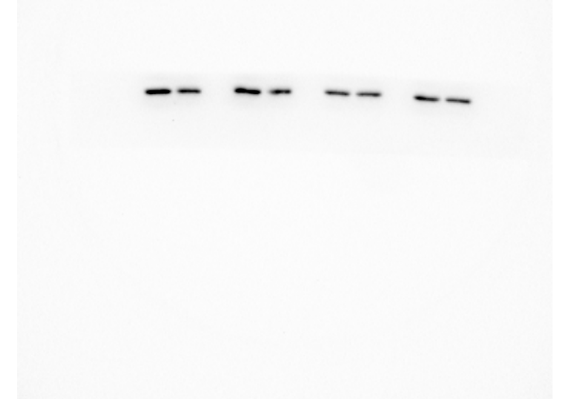

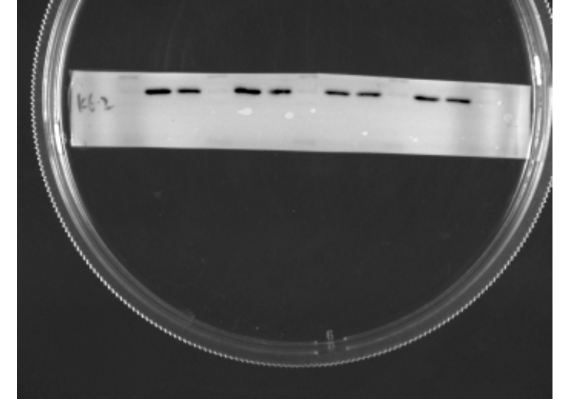

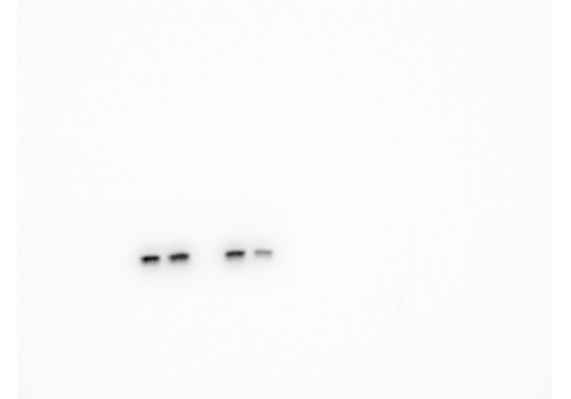
CDK2 (EGI-1)


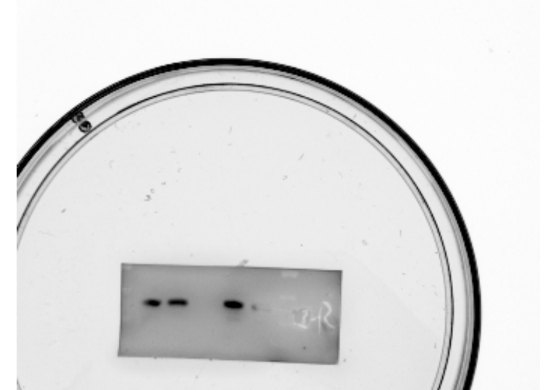

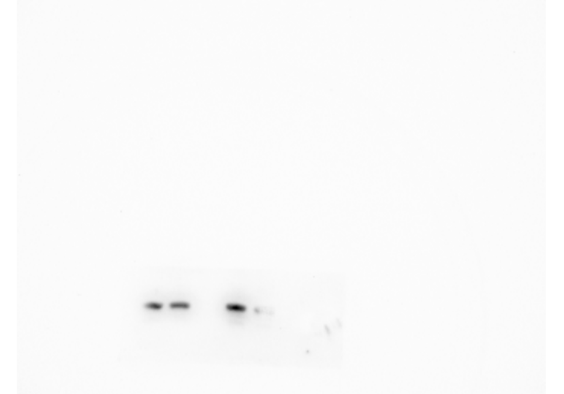
CDK4 (TFK-1)


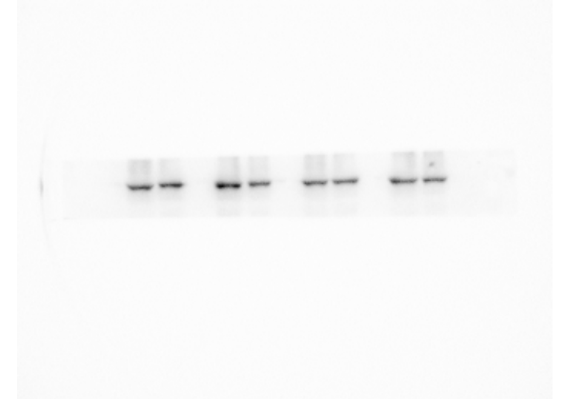

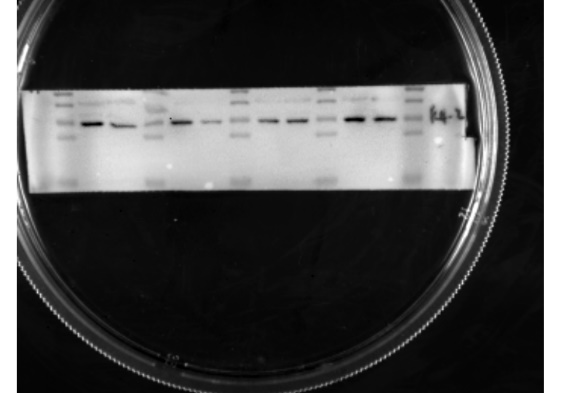
CDK4 (EGI-1)


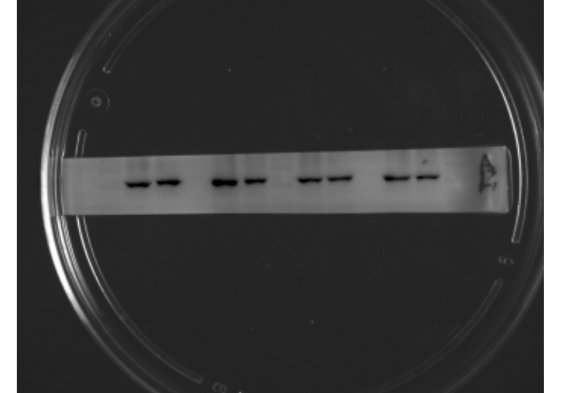

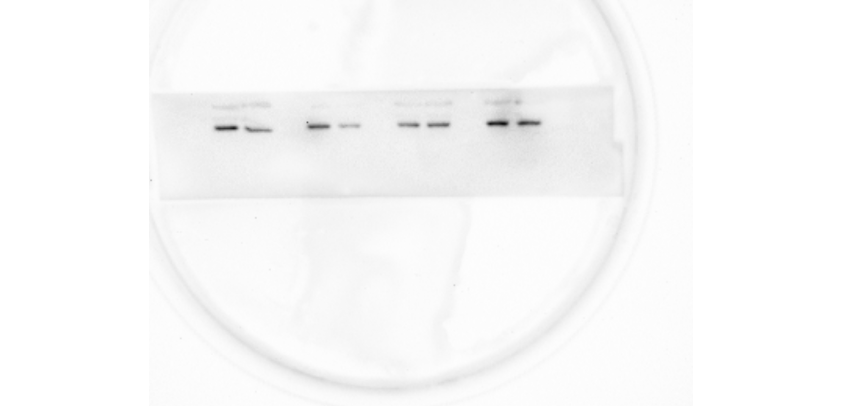
MCM6 (TFK-1)


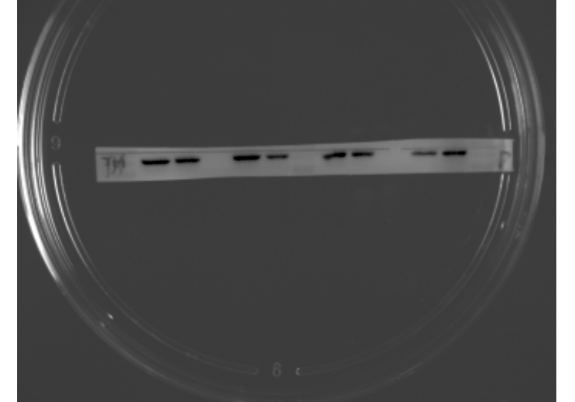

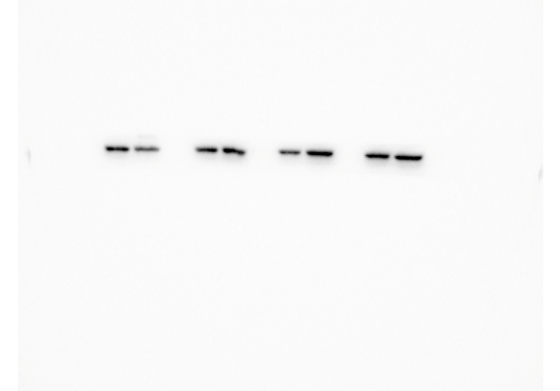
MCM6 (EGI-1)


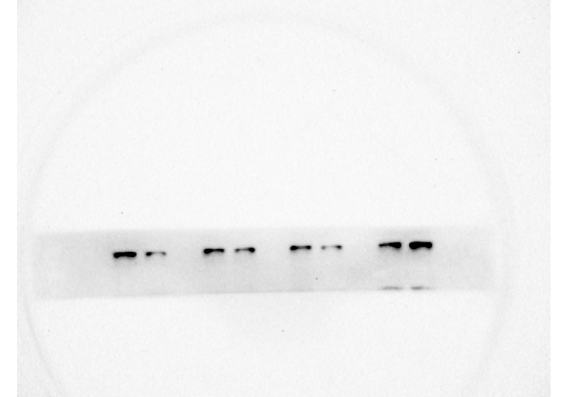

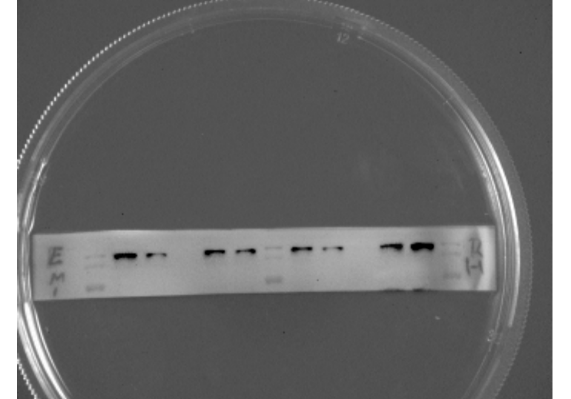
NASP (TFK-1)


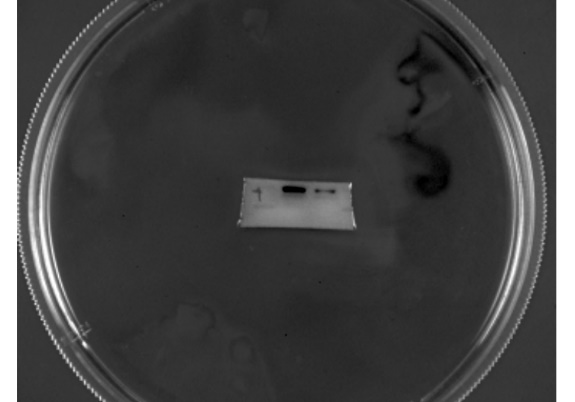

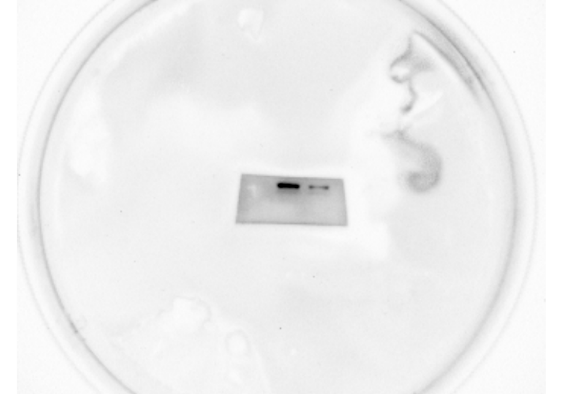
NASP (EGI-1)


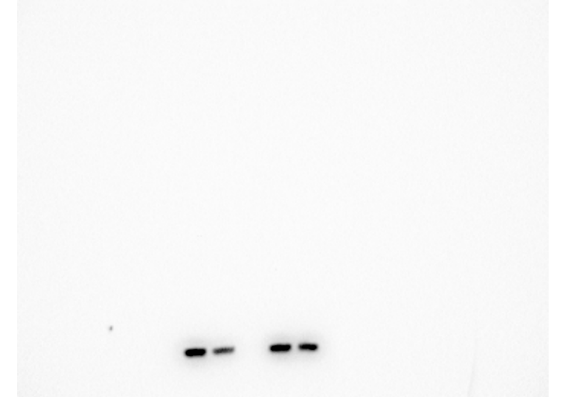

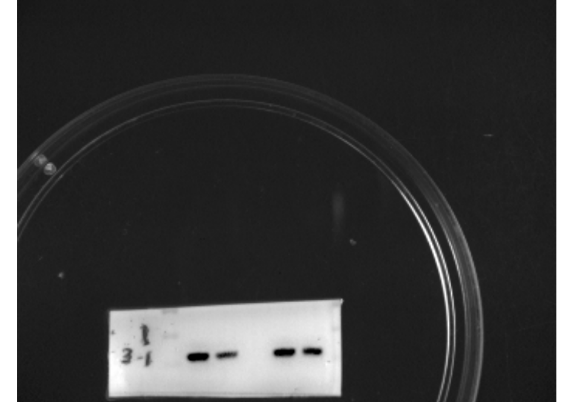
PCNA (TFK-1)


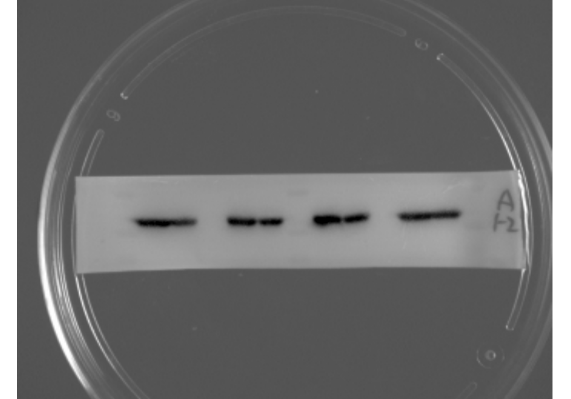

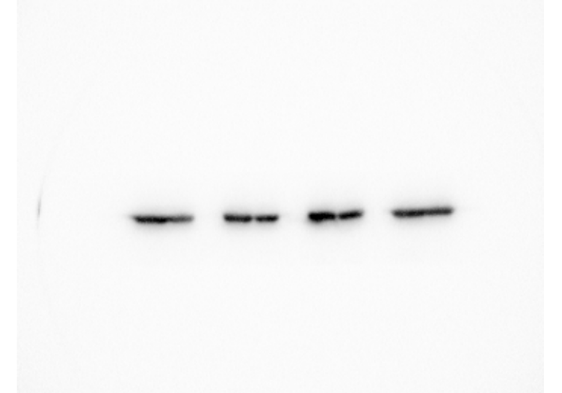
PCNA (EGI-1)


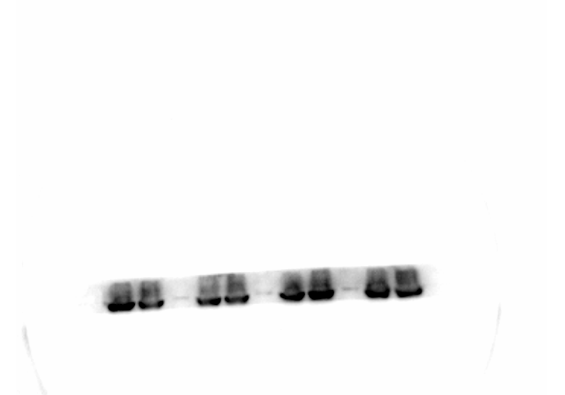

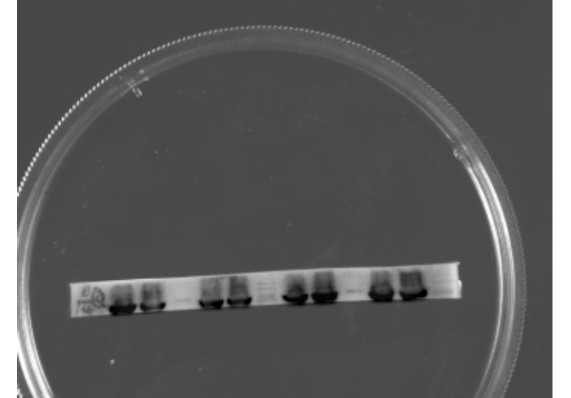
E-Cadherin (TFK-1)


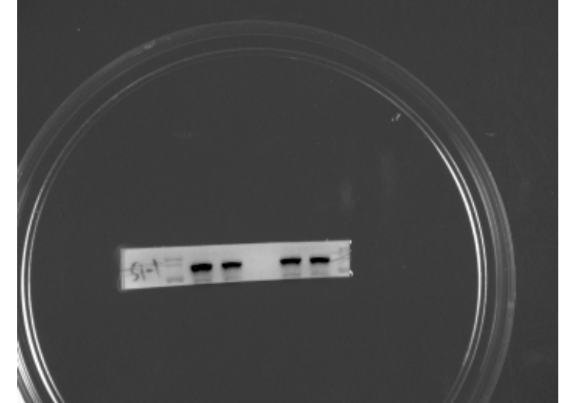
E-Cadherin (EGI-1)


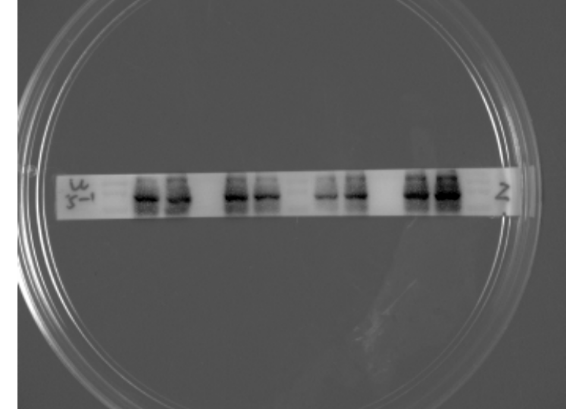

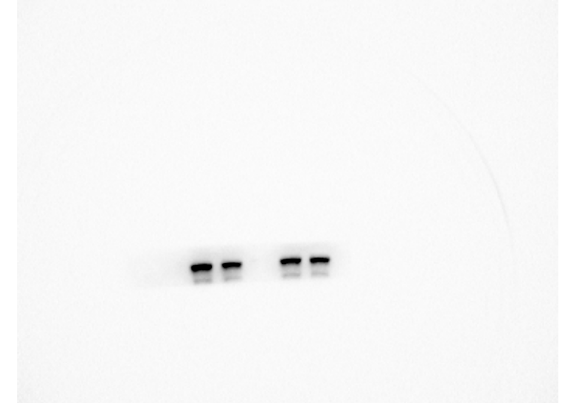
N-Cadherin (TFK-1, EGI-1)


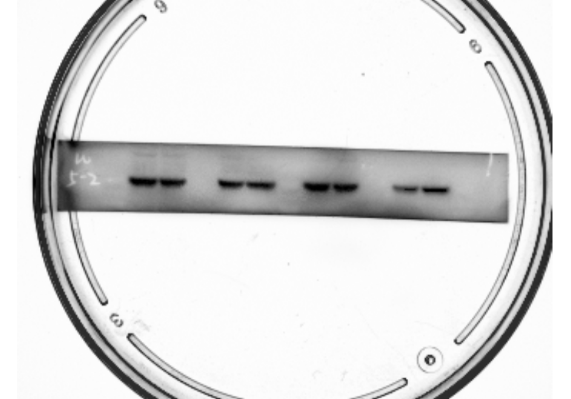

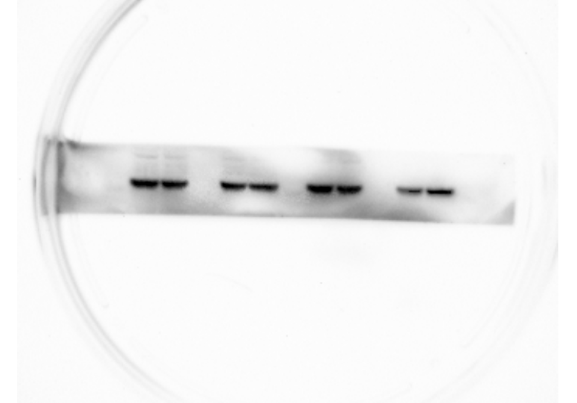

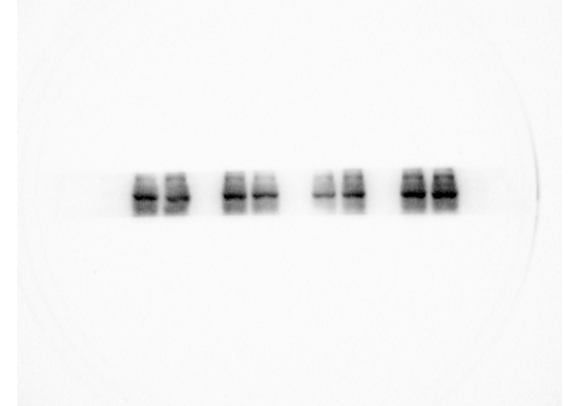
Vimentin (TFK-1, EGI-1)


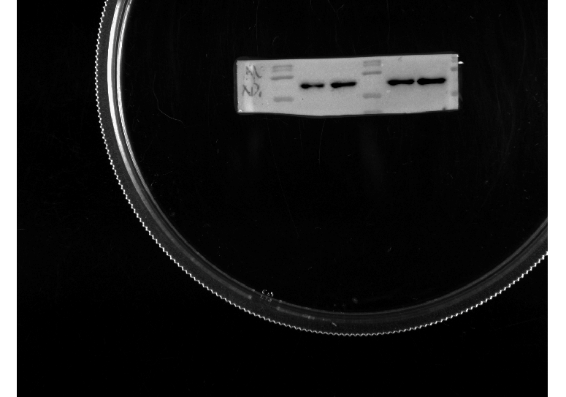

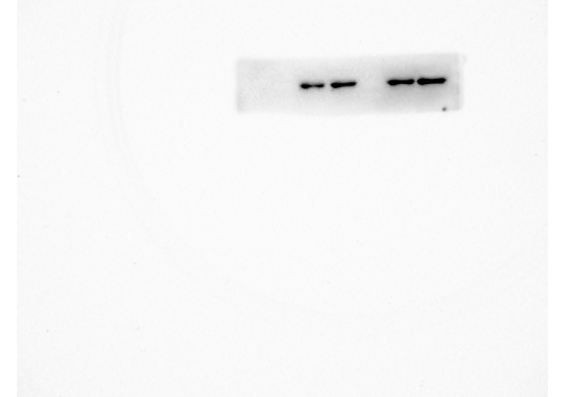
AIFM1 (TFK-1, EGI-1)


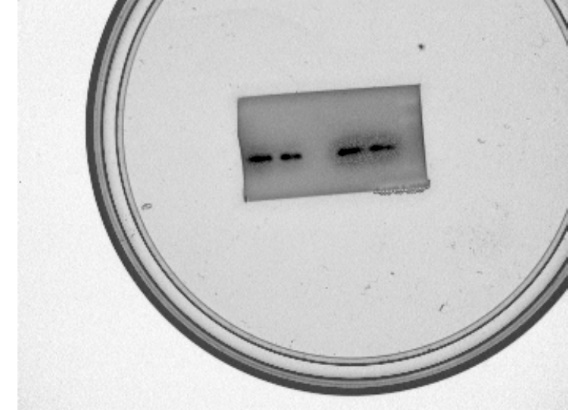
BCL-2 (TFK-1)


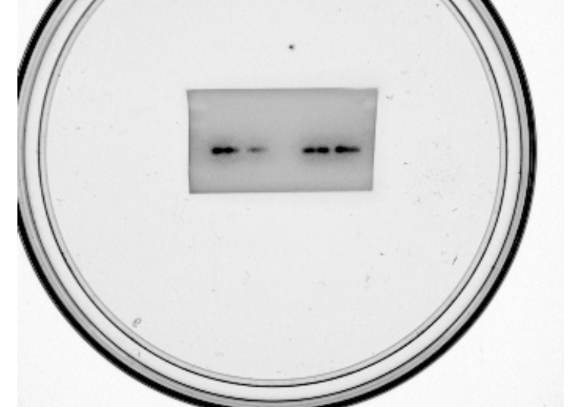
BCL-
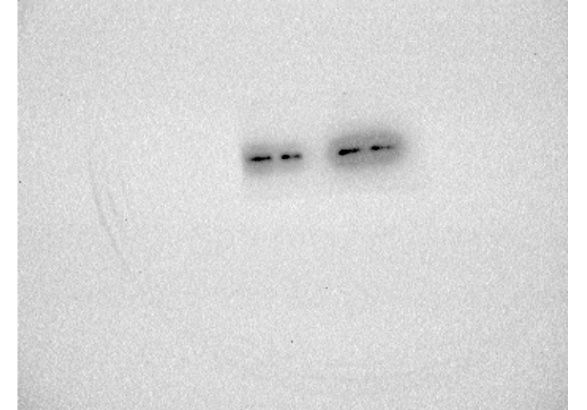
2 (EGI-1)


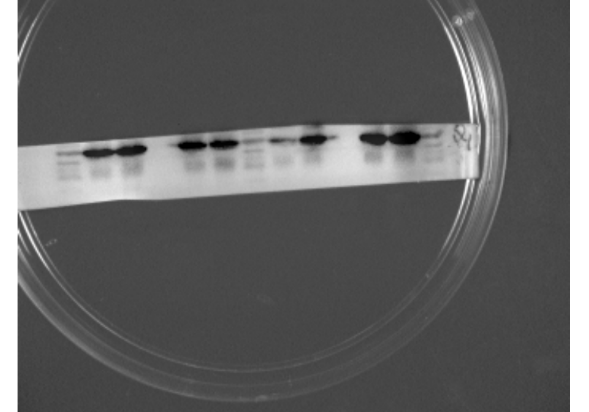

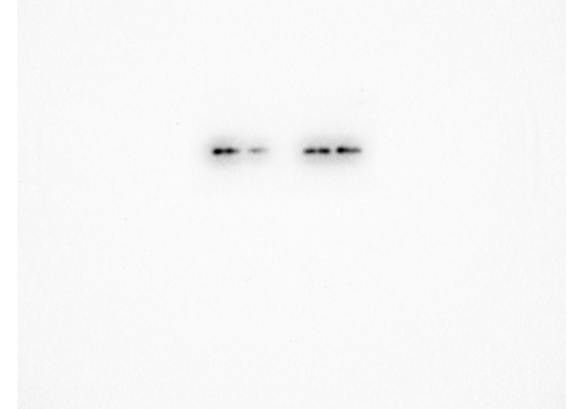
α-Tubulin (TFK-1, EGI-1)


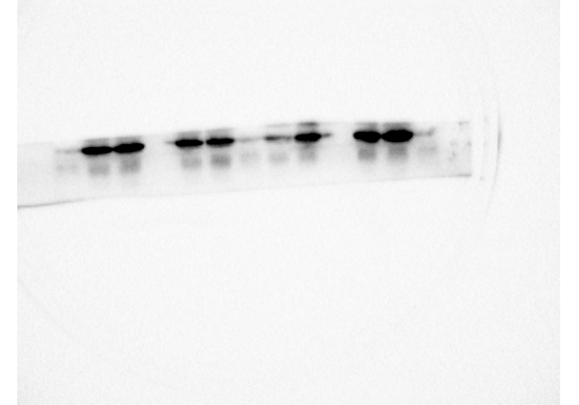


Figure 4

Figure 4J


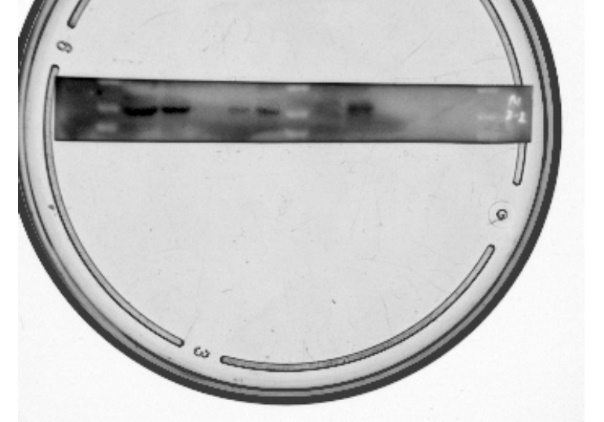
MXD1 (TFK-1, EGI-1)


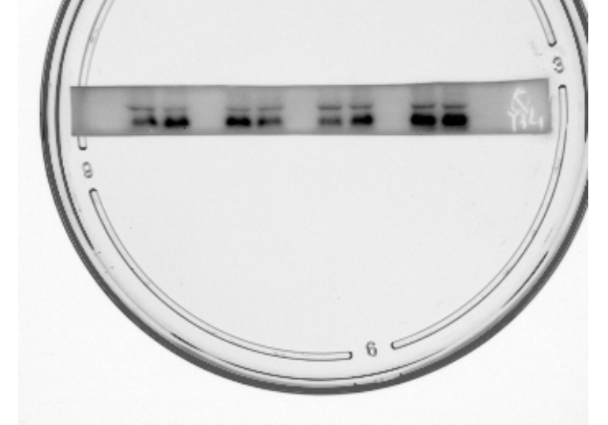

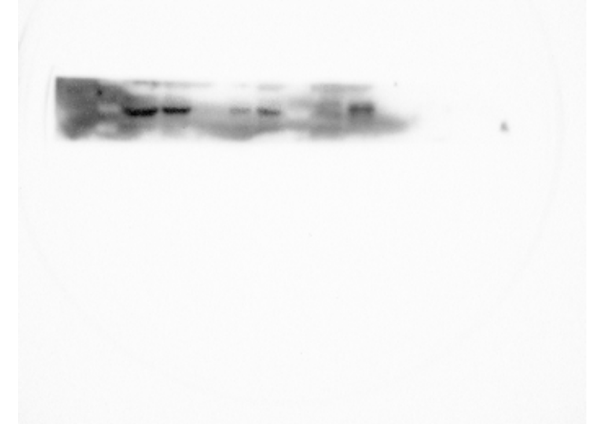
MYC (TFK-1)


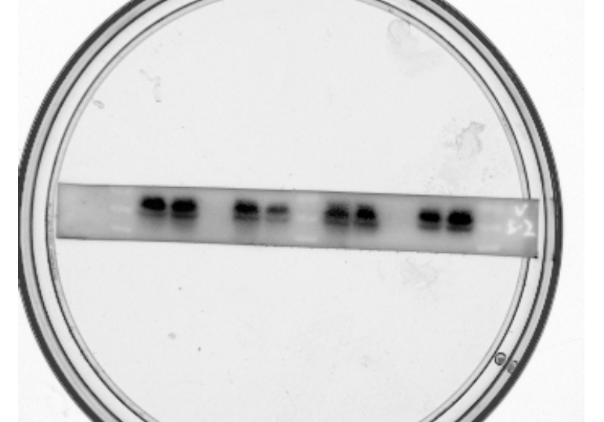

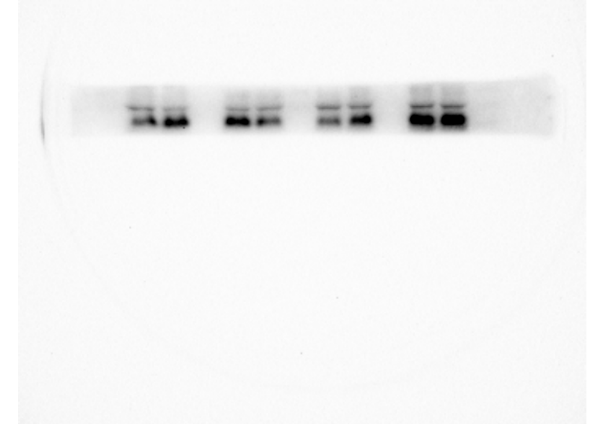
MYC (EGI-1)


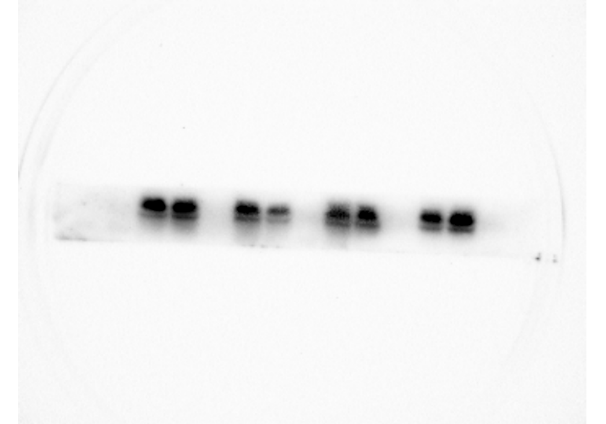


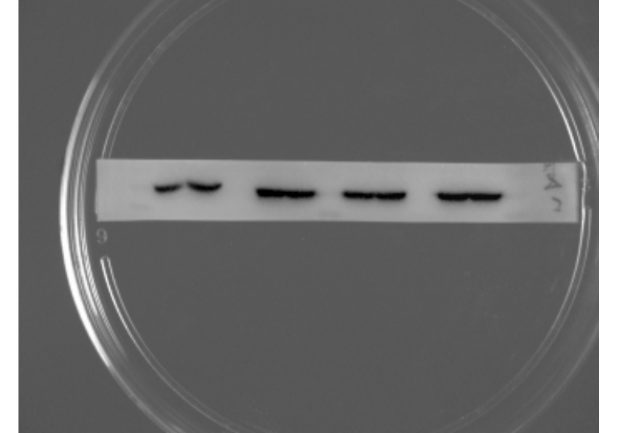
α-Tubulin (TFK-1, EGI-1)

Figure
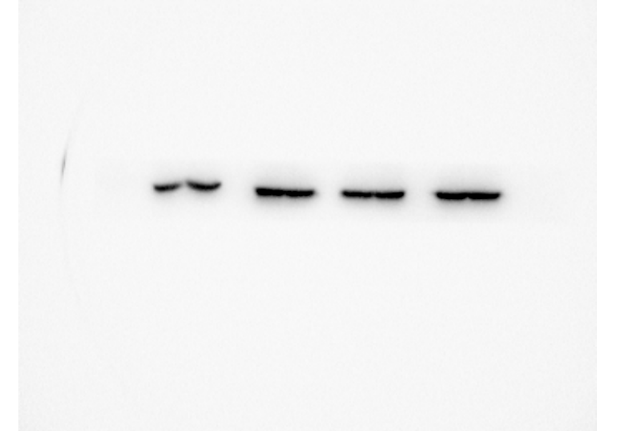
 5

Figure 5B


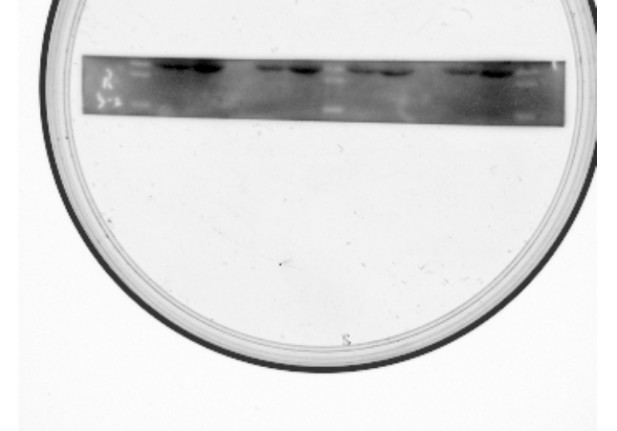
MXD1 (TFK-1, EGI-1)


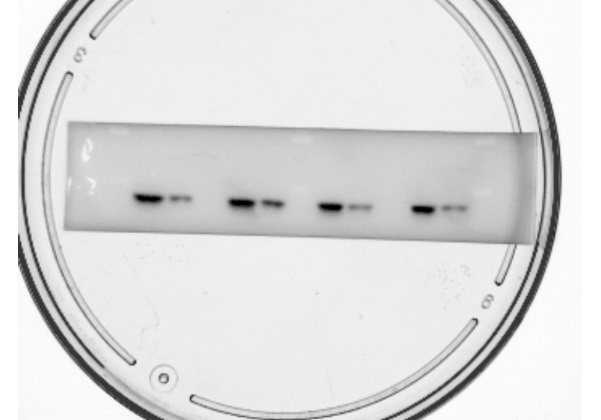

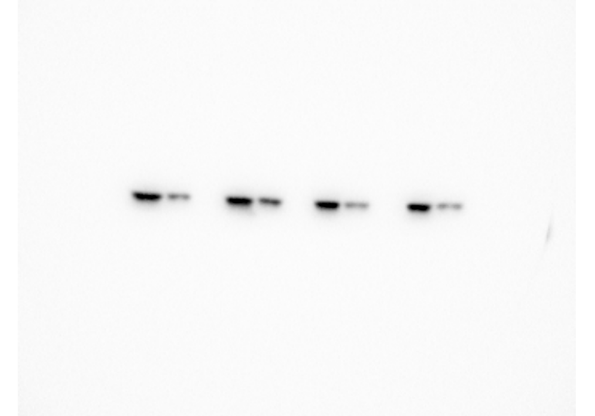

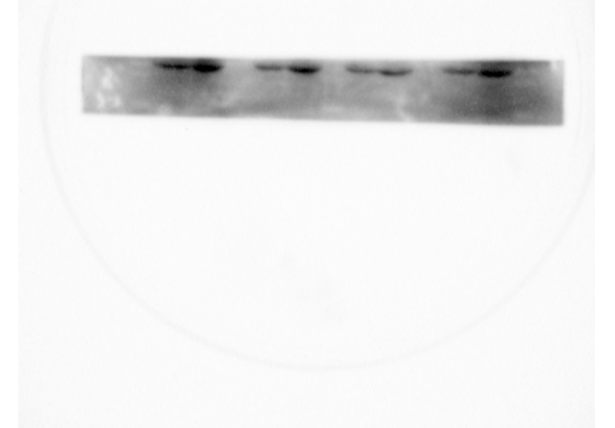
CDK2 (TFK-1, EGI-1)


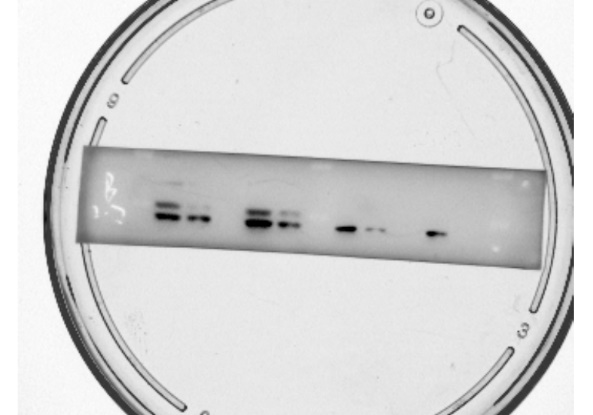

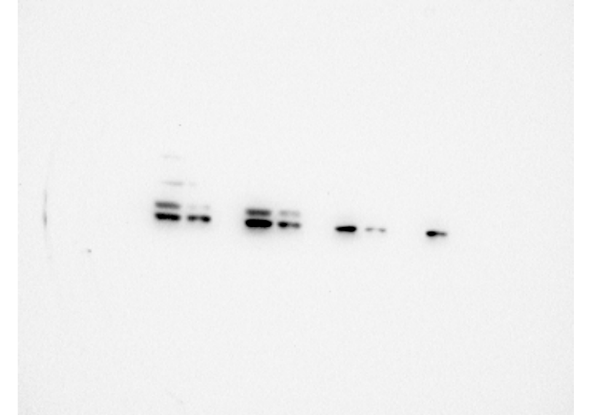
CDK4 (TFK-1, EGI-1)


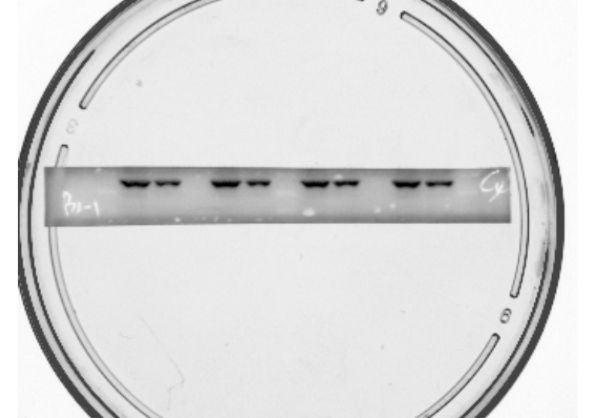
MCM6 (TFK-1, EGI-1)


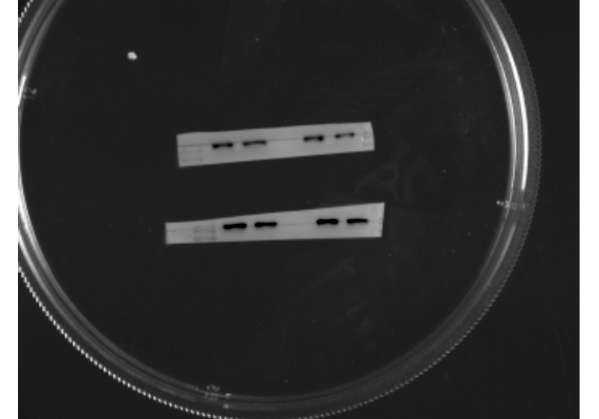

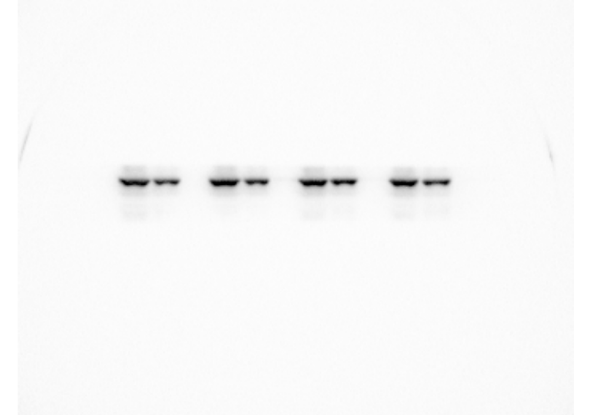
NASP (TFK-1, EGI-1)


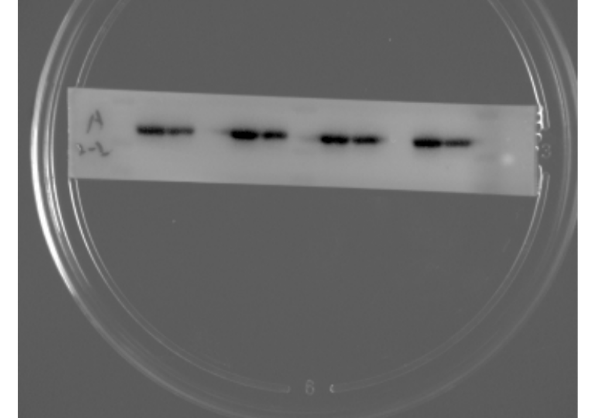

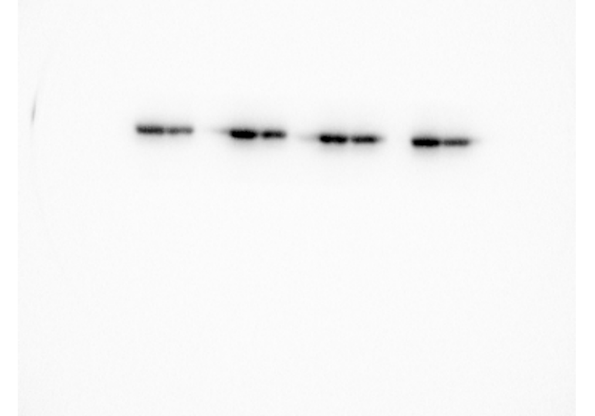
PCNA
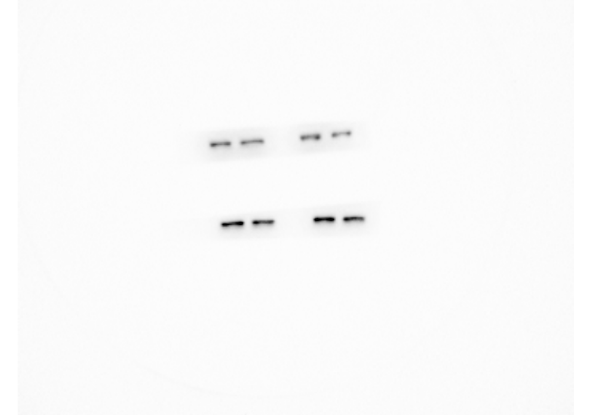
 (TFK-1, EGI-1)


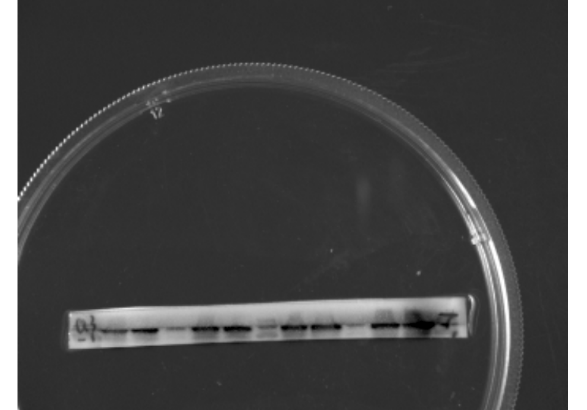
E-Cadherin (TFK-1)


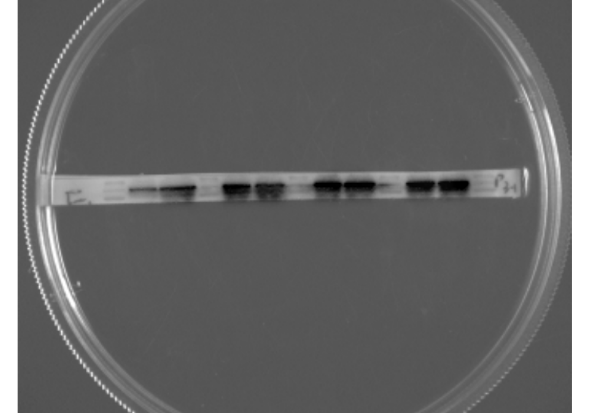

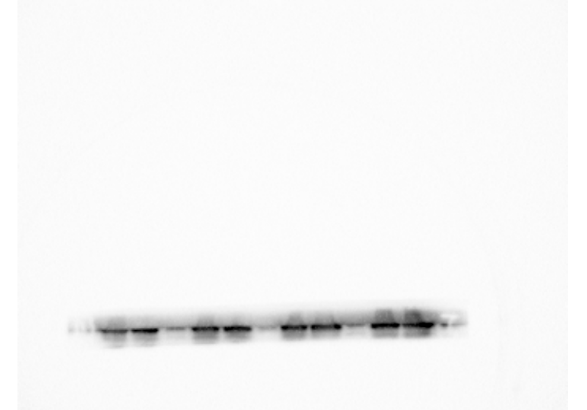
E-Cadherin (EGI-1)


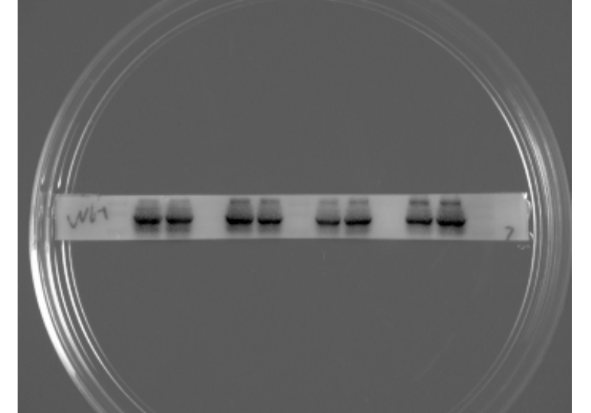

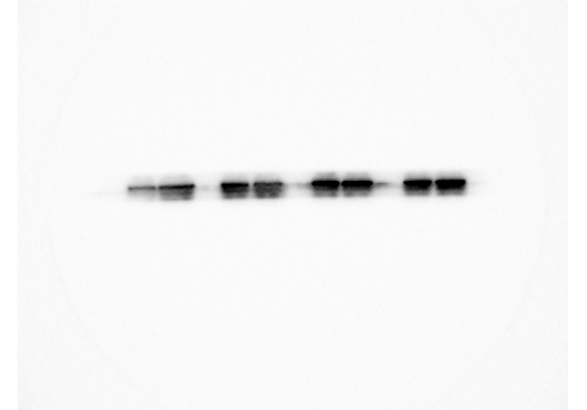
N-Cadherin (TFK-1)


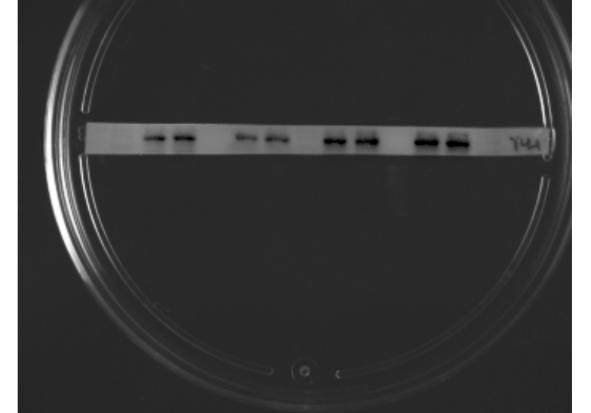

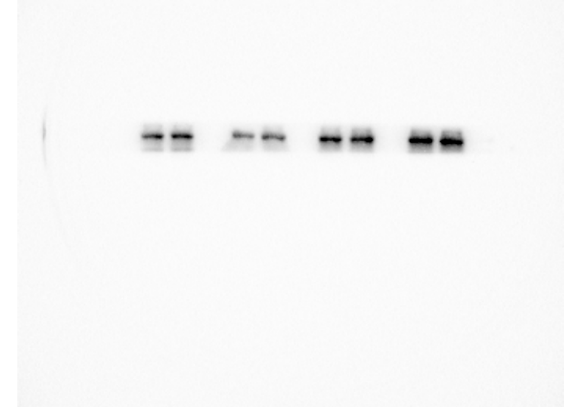

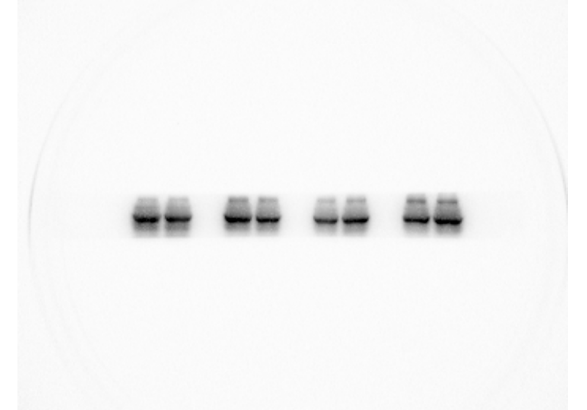
N-Cadherin (EGI-1)


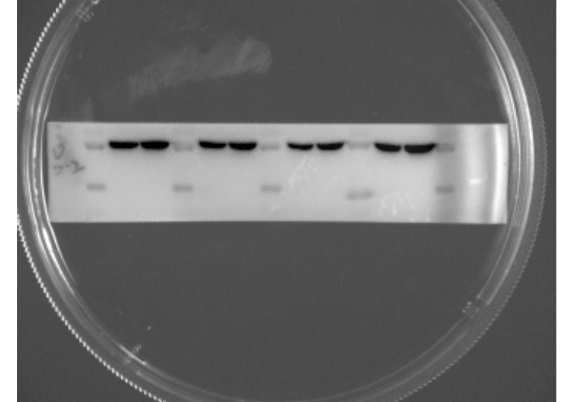
Vimentin (TFK-1, EGI-1)


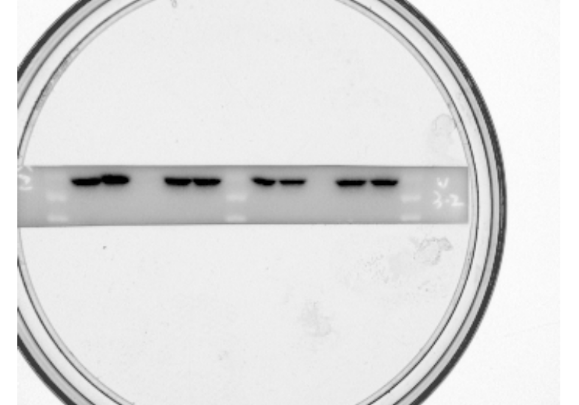

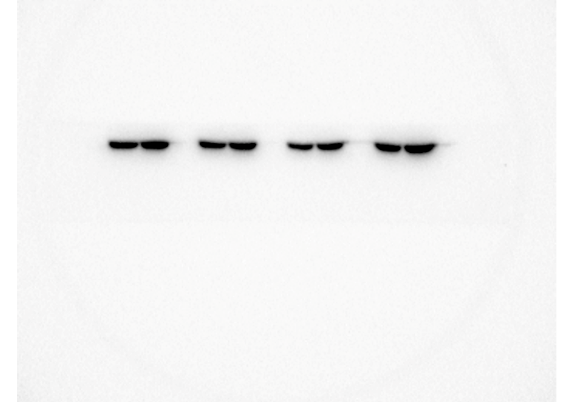
AIFM1 (TFK-1)


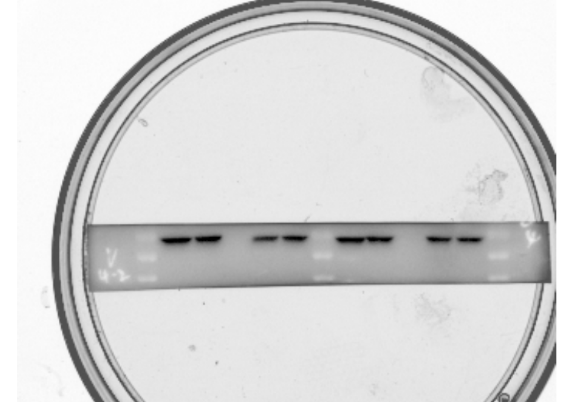

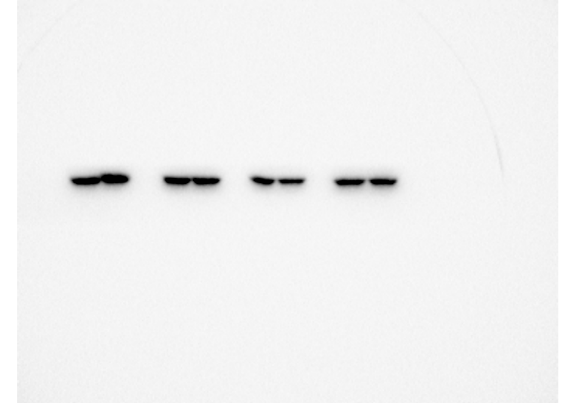
AIFM1 (EGI-1)


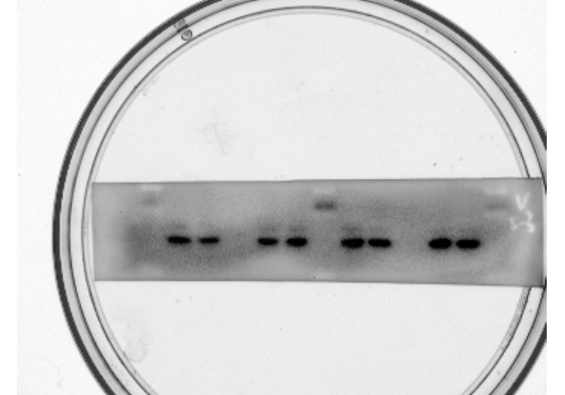

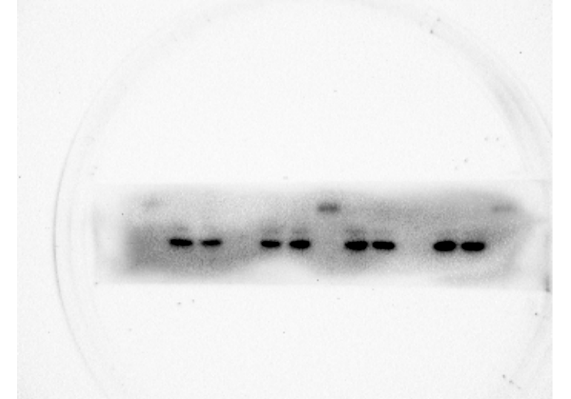

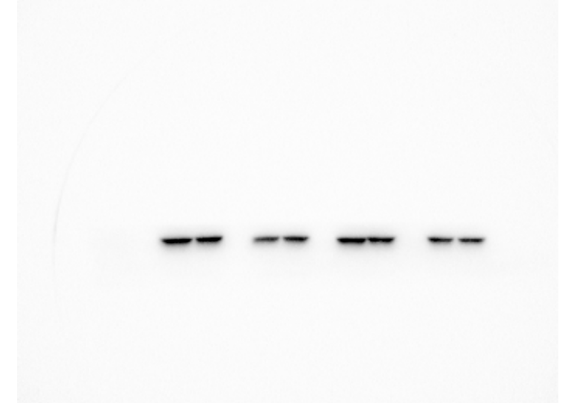
BCL-2 (TFK-1, EGI-1)


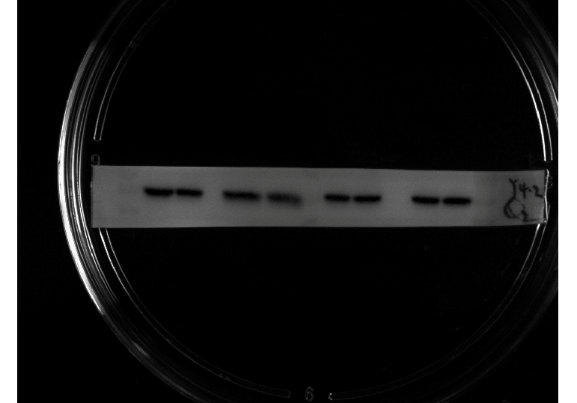
α-Tubulin (TFK-1, EGI-1)


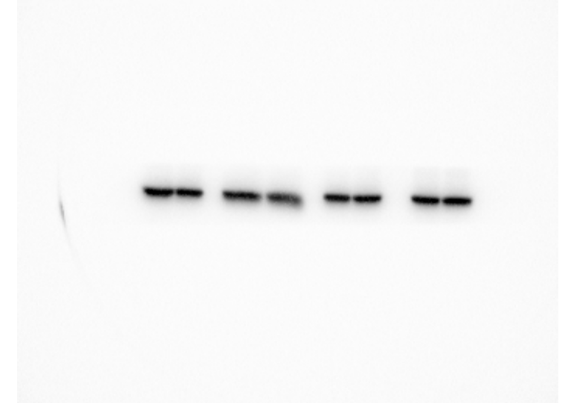


Figure 6

Figure 6B

HOXA5 (TFK-1, EGI-1)

MXD1 (TFK-1)

MXD1 (EGI-1)

CDK2 (TFK-1)

CDK2 (EGI-1)

CDK4 (TFK-1, EGI-1)

PCNA (TFK-1)

PCNA (EGI-1)

NASP (TFK-1)

NASP (EGI-1)

MCM6 (TFK-1)

MCM6 (EGI-1)

α-Tubulin (TFK-1)

α-Tubulin (EGI-1)

Figure 7

α-Tubulin (TFK-1, EGI-1)

p53 (TFK-1)

p53 (EGI-1)

Figure S5

HOXA5(TFK-1, EGI-1)

α-Tubulin (TFK-1, EGI-1)
